# Contrast Agents for Enhanced Bioimaging: A Comprehensive Review

**DOI:** 10.1002/cbic.70418

**Published:** 2026-06-30

**Authors:** Paulo Sérgio Taube, Donald Fernandes, Arthur Abinader Vasconcelos, Hugo de Campos Braga, Alan Kelbis Oliveira Lima, José Arnaldo Santana Costa, Karla Furtado Andriani, Ester da Cunha Meira Santos, Antonio Jorge Silva Araújo Junior, Hauster Maximiler Campos de Paula, Wallace Júnio Reis, Kashif Gul, Nida Wahab, Luis Carlos Resio, Elisa Kawana Leal dos Santos, Márcio Peres de Araujo, Matheus Allen P. da Silva, Juliana Paula da Silva

**Affiliations:** ^1^ Federal University of Western Pará Santarém Brazil; ^2^ Toronto Metropolitan University Toronto Canada; ^3^ Department of Science and Technology Federal University of São Paulo São José dos Campos Brazil; ^4^ Embrapa Agroenergy Brasília Brazil; ^5^ Department of Exact Sciences Santa Cruz State University Ilhéus Brazil; ^6^ Institute of Chemical Sciences University of Peshawar Peshawar Pakistan; ^7^ Department of Chemistry Federal University of Paraná Curitiba Brazil; ^8^ Department of Chemistry Federal University of Santa Catarina Florianópolis Brazil

**Keywords:** contrast agents, metal‐organic frameworks, mesoporous silica materials, multimodal imaging, superparamagnetic iron oxide nanoparticles

## Abstract

Nanotechnology has significantly boosted analytical methods capable of visualizing and characterizing materials at the nanoscale, enabling disease monitoring with high precision. These advances have become particularly relevant in biomedical research, where the demand for enhanced diagnostic tools continues to grow. In this context, contrast agents (CAs) have emerged as essential components in modern imaging techniques, driving efforts toward the development of safer, more efficient, and selective diagnostic materials. The growth in the development of CAs reflects the expansion of applications in imaging modalities. Treatment techniques have benefited from nanotechnology innovations in recent decades, while advancements in imaging modalities demonstrate the demand for next‐generation CAs with enhanced resolution and sensitivity. Nanomaterials play a central role in this progress, offering high surface area, adjustable porosity, structural stability, and easy functionalization, for drug delivery and disease monitoring. Hybrid nanoparticles, including core‐shell systems, clustered architectures, and nanocomposites integrating metallic or magnetic materials with mesoporous silica, zeolites, or metal‐organic frameworks (MOFs), allow control of magnetic relaxivity, X‐ray attenuation, optical response, and biological interactions. Molecular scale and nanosized materials contribute to improved contrast, targeted delivery, and enhanced biocompatibility in bioimaging. Together, these advances highlight the potential of nanotechnology to transform biomedical diagnostics and disease detection.

## Graphical abstract

Contrast agents, especially those that are nanoparticle‐based, are revolutionizing bioimaging by enabling highly sensitive, specific, and early‐stage diagnosis of diseases like cancer, using clinical imaging modalities such as CT, MRI, PET, and SPECT. These advanced materials provide high contrast enhancement, improved tissue penetration, and the ability to target biomarkers at molecular levels, with significantly reduced systemic toxicity compared to traditional agents.

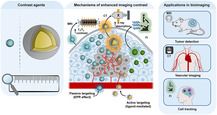

## 1 Introduction

The use of imaging modalities has been important for diagnosis and treatment due to high contrast resolution. Image quality often depends on the use of CAs, with metal‐based compounds being the most widely used in clinical practice. Despite their effectiveness, these CAs have limitations related to their short half‐life, nonspecific distribution, and potential risks from tissue deposition, especially in patients with renal dysfunction [1, 2]. These concerns have driven the development of safer and more efficient alternatives.

Advances in nanoscience have enabled the development of nanoparticles with complex structures and unique physical and chemical properties. Nanomaterials such as superparamagnetic iron oxide nanoparticles (SPIONs), rare earth metals, core‐shell structures, and nanoclusters provide superior light absorption, scattering, fluorescence, and magnetic relaxivity and greater sensitivity in detecting physiological and pathological changes [3, 4, 5, 6]. The ability to design and functionalize these systems allows for active targeting that can result in more precise images and greater diagnostic efficiency.

In recent years, there has been growing interest in hybrid or multimodal agents that combine magnetic properties with fluorescence, CT, or therapeutic potential (i.e., theranostics). These integrated platforms expand diagnostic and intervention capabilities, representing a significant advance within modern nanomedicine [7, 8, 9, 10]. In this context, this review article brings together recent advances in the development, characterization, performance, and applications of CAs for bioimaging, especially those not well studied but holding great promise for future success. For example, zeolites, mesoporous silica materials (MSMs) and MOFs are not well established CAs due to limited amount of in vivo results and studies on toxicity, but can potentially enhance image contrast due to high loading capacity. By integrating experimental findings and current trends, this review seeks to offer a comprehensive overview of the potential of CAs and the challenges that still need to be overcome for their full clinical adoption.

## 2 Methodology

A literature review was conducted to identify recent studies on nanoparticles as CAs for bioimaging, considering publications between January 2021 and November 2025. The Web of Science Core Collection was implemented as the primary database, complemented by searches in Scopus, PubMed/MEDLINE, Embase, and Google Scholar to broaden the retrieval of relevant and highly cited articles. The searches were conducted in English and restricted to original articles and reviews with DOIs, published in journals in the fields of nanomaterials, chemistry, bioengineering, and biomedical imaging. The search strategies used modified terms such as “magnetic nanoparticles,” “SPIONs,” “manganese oxide nanoparticles,” “ferrites,” “contrast agents for magnetic resonance imaging,” “relaxivity,” “T_1_ contrast,” “T_2_ contrast,” “r_1_,” “zeolites,” “mesoporous silica materials,” “contrast agents,” and “r_2_ relaxivity,” ensuring the identification of relevant works on the properties and performance of these nanoparticles in imaging techniques.

The Web of Science filters were adjusted to restrict the publication period, preferentially selecting scientific articles from 2021 to 2025. This approach allowed for the clear gathering and organization of recent literature on materials applied as CAs for bioimaging, offering a broad and up‐to‐date overview of the field. The synthesis was conducted considering general trends, technological advances, different types of materials, and their biomedical applications, without excessively restricting the included studies. Thus, the review sought to integrate relevant contributions published in recent years, highlighting results, experimental approaches, and emerging perspectives, in order to provide a comprehensive and informative overview that reflects the diversity and dynamism of the area.

## 3 Basics of Contrast Agents

Contrast agents are engineered substances that modify imaging signals by changing optical, magnetic, and radioactive properties at local environments. They are mainly classified into organic and inorganic materials, each unique in terms of advantages, limitations, and associated imaging modalities used (Table 1). For example, studies demonstrate that gadolinium loaded nanoparticles can be tuned via structural design to enhance magnetic resonance imaging (MRI) signals and alter T_1_ relaxivity, which is a quantitative measure of how effective a contrast agent is in speeding up T_1_ relaxation of nearby water protons [11]. These agents enhance diagnostic sensitivity by enhancing imaging signals specific to the imaging modality of interest. Structurally modified lanthanide‐based nanoparticles improve image quality by increasing magnetic relaxivity, while their high atomic number results in strong X‐ray attenuation for CT. These nanoparticles also exhibit passive tumor accumulation through the enhanced permeability and retention (EPR) effect, allowing visualization of pathological tissue that would otherwise be impossible to detect. By using positron emission tomography (PET) imaging, dynamic radiotracer methods quantify blood‐brain barrier transport by measuring tracer kinetics, hence enabling detection of alterations in molecular permeability that are not accessible via structural imaging alone [12].

**TABLE 1:** Contrast agents and their unique features for bioimaging. In many cases, hybrid contrast agents with different elements either organic, inorganic or both have been developed to utilize their unique and complementary strengths (i.e., for multimodal imaging). These include for example nanobubbles and metal‐organic frameworks.

Classification	Material types	Advantages	Disadvantages	Imaging techniques
Organic	Lipid based Polymeric Protein based Carbohydrate based	Biocompatible and biodegradable Design flexibility Surface modification and functionalization (e.g., with dyes) Controlled release and response behavior	Complex synthesis Limited thermal and chemical resistance Regulatory and safety concerns	Luminescence imaging Photoacoustic imaging (PAI)
Inorganic	Silica Metal Metal oxide Zeolite	Unique and usually intrinsic electrical, magnetic, and optical properties Tunability of size, structure, and geometry Superior mechanical properties	Stability issues in terms of agglomeration Complex synthesis and high cost Environmental concerns Toxicity problems	Luminescence imaging Photoacoustic imaging (PAI) Magnetic resonance imaging (MRI) Computed tomography (CT) Positron emission tomography (PET) Single photon emission computed tomography (SPECT)

**FIGURE 1:**
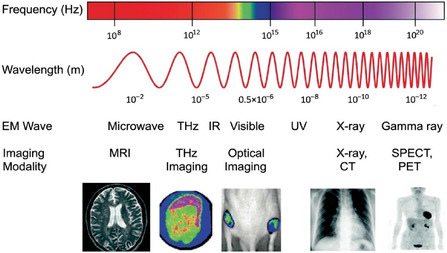
Comparison between the major medical imaging modalities (i.e., MRI, THz imaging, optical imaging, CT, PET, and SPECT). Reproduced with permission from Karthikeyan et al. [13]. Copyright 2021, Springer Nature.

Different imaging techniques apply different physical principles, for example, ultra‐high‐field MRI depends on the magnetic properties of paramagnetic elements, while computed tomography (CT) relies on the strong X‐ray attenuation produced by high atomic number (Z) elements (Figure 1). Nanoparticle‐based systems have become central to contrast agent design because their composition, size, and surface architecture can be systematically controlled.

## 4 Historical Overview of Contrast Agents

This section provides a comprehensive overview of CAs used in medical imaging, tracing their development from early conventional compounds to modern advanced systems. It discusses the historical background and evolutionary milestones of CAs, highlighting key advances in chemical design, safety, and imaging performance. Particular emphasis is placed on the rise of nanotechnology‐based CAs and the emergence of MOFs as innovative platforms in diagnostic imaging, reflecting current trends aimed at improving sensitivity, specificity, and multifunctionality in biomedical diagnostics.

### 4.1 Early MRI Contrast Agents

In the 1980s, development of MRI CAs gained momentum with the introduction of gadolinium(III) chelates, especially Gd‐DTPA. Experimental studies demonstrated that chelating Gd^3+^ with diethylenetriaminepentaacetic acid (DTPA) produces a strongly paramagnetic and kinetically stable compound capable of reducing proton relaxation times even at very low concentrations. In vivo, Gd‐DTPA behaves as a vascular agent, remaining largely within the bloodstream immediately after injection, rapidly accumulating in the kidneys and undergoing predominantly renal excretion, with more than 90% eliminated within 24 h and only about 2% of the injected dose remaining after 120 min. This favorable biodistribution contrasts mainly with ionic GdCl_3_, accumulating extensively in the liver and spleen in protein bound or colloidal forms, yielding limited relaxation effects. These findings established Gd‐DTPA as a well‐tolerated and a highly effective vascular MRI contrast agent, hence laying the foundation for its clinical adoption [14]. Early clinical studies by Carr and later by Runge confirmed strong tumor enhancement and established gadolinium chelates as standard T_1_ weighted MRI CAs [15]. Although long considered safe, certain gadolinium‐based contrast agents (GBCAs) were later linked to nephrogenic systemic fibrosis (NSF) in patients with severe renal impairment [16]. Later, awareness and updated guidelines have significantly reduced number of new NSF cases.

### 4.2 CT Contrast Evolution

**FIGURE 2:**
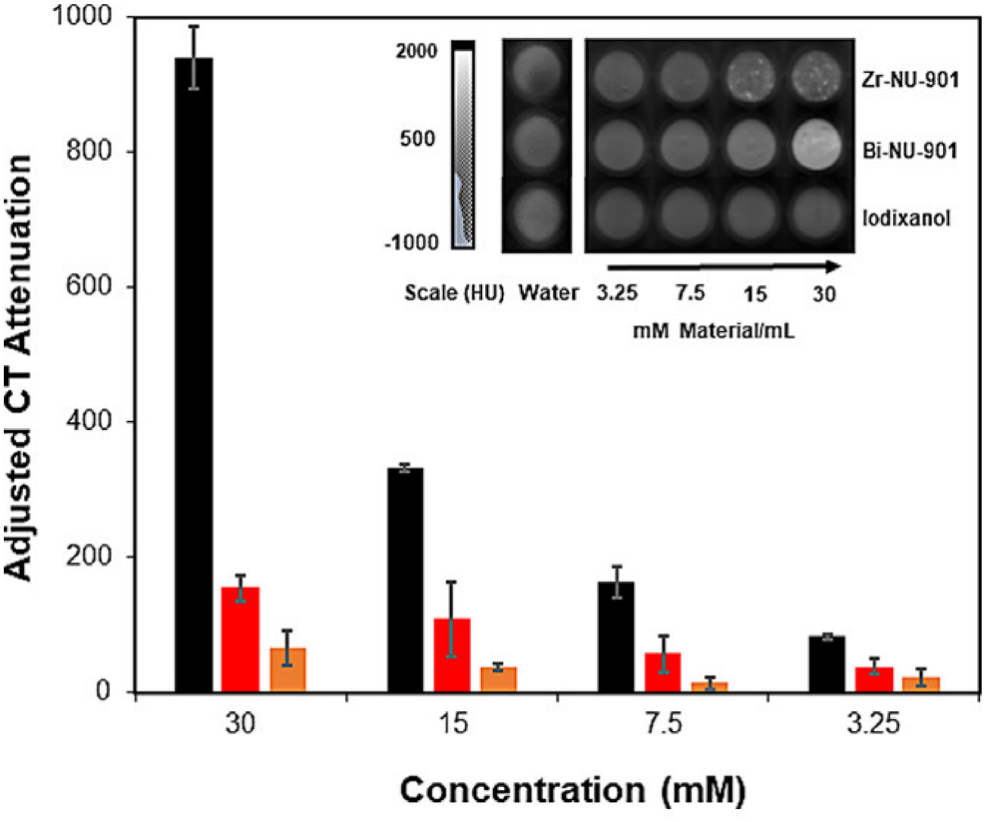
Adjusted CT attenuation values for Bi‐NU‐901 (black), Zr‐NU‐901 (red), and the commercial contrast agent iodixanol (orange) measured across varying concentrations (3.25–30 mM). Bi‐NU‐901 consistently exhibits markedly higher attenuation, especially at 30 mM, demonstrating its superior X‐ray contrast potential (reproduced from Robison et al. [26], Copyright 2019, ACS).

CT relies on X‐ray attenuation, with contrast agent advancements addressing issues with soft tissue similarity in contrast [17]. Early development included low osmolar nonionic iodinated agents, which provided equivalent diagnostic quality with significantly fewer cardiovascular and physiological side effects than older high osmolar ionic media, hence leading to their rapid clinical adoption. More recent research focuses on nanoparticle‐based CT agents that are fabricated for strong X‐ray attenuation, tunable size plus surface chemistry, and improved biodistribution [18, 19, 20]. Metallic nanoparticles highlight how composition and surface modification influence imaging sensitivity and targeted delivery [21, 22]. Fucoidan functionalized HfO_2_ nanoparticles, for example, have shown efficient attenuation and can be used for targeted plaque imaging using spectral photon counting CT [23]. Likewise, small molecule bismuth chelates and bismuth‐based nanomaterials have demonstrated superior CT contrast and effective imaging of kidneys, bladder, and tumors in vivo as compared with standard iodinated media [24, 25]. For instance, the bismuth‐based MOF, Bi‐NU‐901, featuring a *scu* topology and ∼11 Å pores, has been synthesized as a high‐performance contrast agent for X‐ray CT. The framework is constructed from Bi_6_ nodes and TBAPy linkers, with structural characterization (i.e., PXRD, STEM, and DFT pore analysis). Owing to its high density of nontoxic heavy atoms, Bi‐NU‐901 exhibits exceptional CT contrast, providing approximately sevenfold higher intensity than an isostructural zirconium MOF and nearly fourteenfold greater contrast than a commercial CT agent (Figure 2) [26].

### 4.3 Rise of Nanotechnology and MOFs in Diagnostics

In the 1980s and 1990s, nanotechnology expanded MRI capabilities with SPIONs [4]. Weissleder's research on ultrasmall SPIONs (<10 nm) demonstrated long circulation, extensive uptake by the reticuloendothelial system, and strong T_2_/T_2_* contrast effects, establishing SPIONs as a new diagnostic class [27]. Afterwards, MOFs emerged as modular systems for combined imaging and drug delivery [28].

Early studies demonstrated that Fe(III)‐carboxylate MOFs could be functionalized with dyes or therapeutic payloads, supporting controlled release and optical or MR‐based visualization [29]. Subsequent research confirmed their high drug loading capacity, biocompatibility, and potential as multifunctional theranostic carriers, that distinguish MOFs from traditional chelates and inorganic nanoparticles [30].

## 5 Phenomena Responsible for Contrast

This section addresses the fundamental phenomena responsible for image contrast in diagnostic techniques, with a primary focus on X‐ray and magnetic resonance based modalities. It explores the mechanisms underlying contrast generation in X‐ray, CT and MR imaging, including X‐ray attenuation and magnetic relaxation processes and the role of the photoelectric effect. It further examines the contribution of transition metal complexes as CAs, emphasizing their electronic structure and interaction with ionizing radiation.

### 5.1 X‐Ray and CT Contrast Mechanisms

X‐ray and CT image contrast is basically governed by how strongly different materials attenuate X‐rays. Materials containing high atomic number (high Z) elements, especially gold (Au), hafnium (Hf), and iodine (I), consistently exhibit superior attenuation and therefore higher contrast in CT imaging. Multiple research studies report that gold nanoparticles (AuNPs) produce very strong X‐ray attenuation and CT values (i.e., in vitro and in vivo), with better contrast to noise ratios (CNRs) across all tested energies, compared to conventional iodinated agents [31, 32]. Smaller spherical AuNPs have higher X‐ray attenuation than larger ones and AuNPs with larger aspect ratios (i.e., length to its width or diameter) exhibit greater effect on X‐ray attenuation. Smaller AuNPs provide a larger total surface area, leading to greater interaction with incident photons. The greater X‐ray attenuation of AuNPs of larger aspect ratios could be associated with their higher gold content compared to smaller ones. Surface coating (e.g., using polyethylene glycol, PEG) of AuNPs declines X‐ray attenuation as a result of limiting the aggregation of NPs. The presence of molecules in the form of double layer on the surface of AuNPs can facilitate side to side ordering of the AuNPs, which results in their behavior like larger structures with larger X‐ray attenuation. Overall, smaller spherical AuNPs can be suggested as a better alternative to conventional CAs for CT imaging [33]. Size, morphology and surface chemistry are also important for the application of gold nanostructures, in radiation dose enhancement, where NPs with high X‐ray attenuation are applied.

Besides this, hafnium oxide (HfO_2_) nanoparticles are also repeatedly identified as strong CT attenuators due to the high atomic number (Z = 72) of hafnium. Their nanoparticles are superior with comparable CT contrast to Au at tube potentials above 80 kVp and significantly greater contrast than iodine. Their strong CT contrast arises from the favorable position of the hafnium K‐edge (≈65 keV), which increases attenuation in clinical CT energy ranges [17].

### 5.2 Fundamentals of MRI Contrast

MRI contrast depends fundamentally on how hydrogen protons behave in different microenvironments when subjected to magnetic fields and RF excitation. Once protons are aligned with the static magnetic field, RF pulses cause excitation, and the subsequent relaxation processes determine signal intensity. The longitudinal relaxation time (T_1_) reflects the rate at which protons realign with the external field, whereas the transverse relaxation time (T_2_) describes the loss of phase coherence among spins due to environmental interactions [34].

**FIGURE 3:**
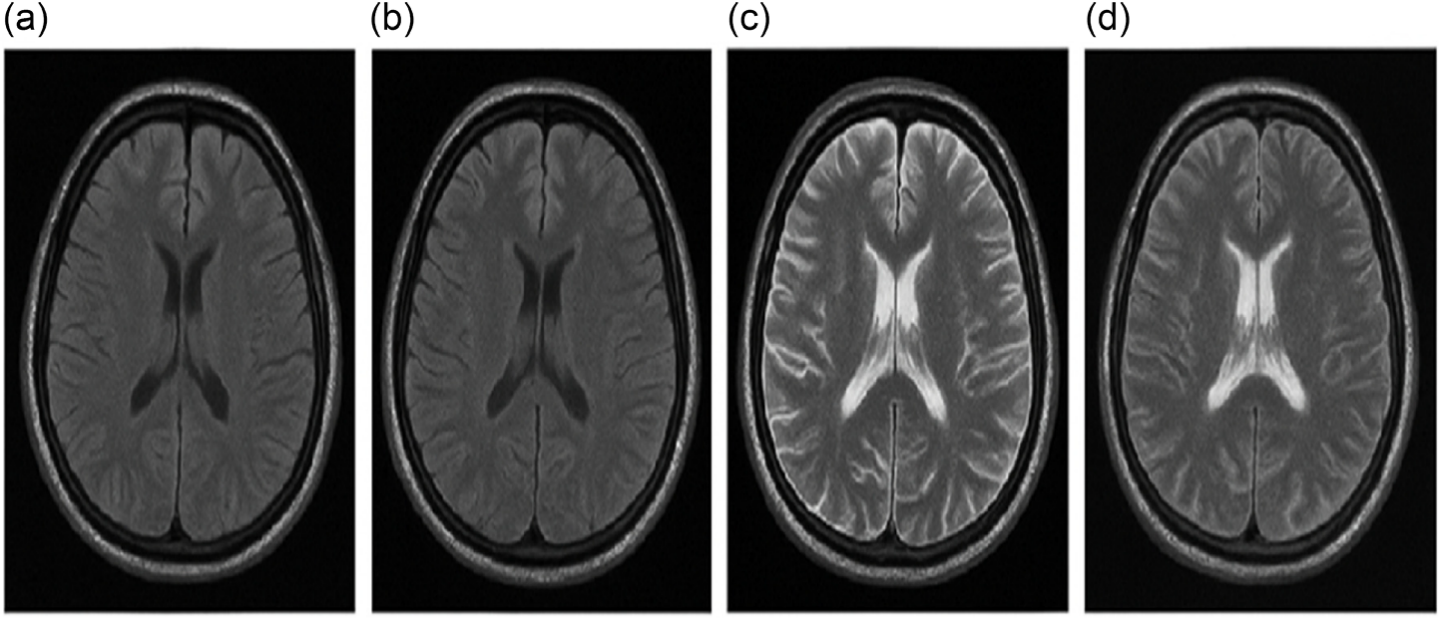
Representative MRI contrast mechanisms. Comparison of (a) MRI without contrast agent, (b) T_1_‐positive contrast, (c) T_2_‐negative contrast, and (d) hybrid T_1_–T_2_ dual‐mode contrast enhancement, illustrating signal brightening, darkening, and mixed‐mode effects commonly observed in nanomaterial‐based agents.

The diagnostic utility of MRI arises from these relaxation differences. T_1_‐weighted sequences are typically used to visualize anatomical structures, such as fat‐rich structures, while T_2_‐weighted sequences are important in detecting edema, inflammation, demyelination, and other pathophysiological changes associated with increased tissue water content (Figure 3). When used together, these sequences provide a more complete diagnosis, allowing for the differentiation of diseases and the identification of pathological changes with greater precision [35, 36].

Because small changes in proton relaxation can represent clinically relevant differences, the use of CAs becomes essential. CA‐induced relaxation enhancement results from local magnetic perturbations caused by paramagnetic or superparamagnetic species. T_1_ agents increase r_1_ relaxivity and produce hyperintense signals, whereas T_2_ agents increase r_2_ relaxivity and promote signal darkening in tissues with high accumulation of nanoparticles [37].

The relaxivity values r_1_ and r_2_ quantify the efficiency of a contrast agent in accelerating proton relaxation. Metal nanoparticle (MNP) relaxivity depends on multiple factors: i) core size and crystallinity, which influence magnetic moment; ii) surface coatings, determining hydrophilicity and water accessibility; iii) aggregation state, which modifies diffusion‐related dephasing; iv) hydrodynamic diameter, affecting circulation time; v) coating thickness, which modulates proton proximity; vi) magnetic field strength, with higher fields favoring different relaxation pathways, and vii) spin‐electron coupling, which differs between Mn^2+^, Fe^3+^, and ferrite structures. In general, the properties of magnetic nanoparticles are closely related to their particle sizes [38]. Neither longitudinal relaxation rate r_1_ nor transverse relaxation rate r_2_ change monotonously with the particle size. However, smaller magnetic nanoparticles tend to have larger r_1_ relaxation rates. The effect of CAs on T_1_ relaxation mechanism is mainly due to the direct interaction of hydrogen nucleus in water molecules and the metal ions in magnetic nanoparticles to shorten the T_1_ relaxation time and not directly related to magnetic field inhomogeneity. It is related to the number of coordination water molecules, the exchange rate of water molecules, and the rotation time of complexes. The smaller the particle size, the larger the specific surface area S/V, and the easier the interaction between unpaired electrons of metal ions in nanoparticles and water electrons, leading to larger r_1_ relaxation rate. As regards to the r_2_ relaxation rate, it is directly related to the magnetic field inhomogeneity caused by magnetic nanoparticles. The magnetization induced by magnetic nanoparticles in the magnetic field can be considered to be directly affecting the magnetic field inhomogeneity. The induced magnetization of magnetic nanoparticles is directly related to the magnetic moment. Therefore, if the saturation magnetization of magnetic particles is constant, the larger the size of magnetic particles, the greater the induction magnetization and the larger the r_2_ relaxation rate. Typical T_1_ agents require small cores, high r_1_ values, and low r_2_/r_1_ ratios (≤4), while T_2_ agents rely on large magnetic moments and high r_2_ values, with high r_2_/r_1_ ratios (i.e., often exceeding 10) [39]. The shape effect of nonspherical magnetic nanoparticles may generate a larger area of effective spin perturbation than that of spherical magnetic nanoparticles with equivalent saturated magnetization values, leading to enhanced relaxivity. In addition, higher degree of magnetic spin order within crystals leads to greater T_2_ relaxivity. The T_2_ relaxivity is correlated to the anchoring nature of surface molecules on magnetic nanoparticles, due to the alternation of surface spin canting effect through specific coordination. The binding affinity of anchoring ligands (e.g., used for functionalization of NPs) is strongly correlated to the magnetic moment of magnetic nanoparticles. In particular, higher binding affinity of anchoring ligands leads to lower magnetic moment of the resulting magnetic nanoparticles, in which π‐π and *p*‐π conjugations between the anchoring ligands and magnetic nanoparticles are identified as the main reason of enhancing the magnetization effect of NPs. This has provided new strategies to customize the magnetic properties and MRI performance of NPs through alternating the chemical structure of surface ligands [40]. The effect of surface coating thickness on the relaxivity is determined by two competing factors: the physical exclusion of water protons away from magnetic nanoparticles and the increased residence time of water molecules within the polymer layer. Many polymers and mesoporous silica on the surface of magnetic nanoparticles permit optimal access of water molecules to the surface, which boost both the T_1_ and T_2_ relaxation enhancement effects.

T_1_‐weighted MRI relies on CAs capable of efficiently shortening longitudinal relaxation times, thereby producing hyperintense signals in tissues where they accumulate. Although gadolinium‐based small‐molecule chelates such as Gadovist, Dotarem, and MultiHance remain the clinical standard, concerns related to NSF, residual brain deposition, and instability of Gd^3+^ complexes under acidic or competitive‐chelation conditions have stimulated the search for safer alternatives [41, 42, 43]. In this context, magnetic nanoparticles engineered for positive contrast have emerged as a rapidly expanding category, particularly ultrasmall SPIONs and manganese‐based nanostructures, which offer improved safety profiles, higher ionic relaxivity, and versatile surface chemistry for targeted or multimodal imaging [44, 45].

T_2_‐weighted MRI relies on CAs capable of accelerating spin–spin dephasing, leading to localized hypointense signals in tissues where the agent accumulates. Magnetic nanoparticles used for T_2_ contrast are typically SPIONs, which exhibit strong magnetic susceptibility and high r_2_ values due to their ability to generate local field inhomogeneities. SPIONs remain the most clinically validated class of magnetic nanoparticle CAs, with several formulations approved or evaluated in clinical settings.

Development of dual‐mode T_1_–T_2_ magnetic nanoparticles constitutes one of the most rapidly advancing areas in contrast agent engineering, offering synergistic enhancement of MRI performance by integrating the bright‐signal characteristics of T_1_ agents with the negative contrast capabilities of T_2_ agents. This combination allows for self‐validating diagnostic outputs, reduces interpretation ambiguity, and significantly improves lesion localization and differentiation, particularly in complex tumor microenvironments where single‐mode agents may generate misleading results [46].

The design of T_1_–T_2_ hybrid agents requires a delicate balance between competing relaxation mechanisms. T_1_ enhancement demands efficient proton–electron dipolar coupling and minimal magnetic susceptibility effects, while T_2_ contrast relies on strong local field inhomogeneities generated by high magnetization cores. Achieving simultaneous functionality therefore involves advanced control over particle composition, core–shell architectures, and stimuli‐responsive behavior.

### 5.3 X‐Ray Attenuation, Photoelectric Effect, and Magnetic Relaxation

The photoelectric effect influences X‐ray density logging. Analyzing how Compton scattering and photoelectric absorption vary with energy in geological formations makes it clear that low energy X‐rays are specifically sensitive to photoelectric interactions, which in turn distort density estimates. On the other hand, higher energy γ‐rays, such as those emitted by Cs‐137, are far less affected. A correction strategy that uses count data from both a density sensitive window and a lithology sensitive energy window can effectively compensate for this error, as confirmed through Monte Carlo simulations. This approach can achieve density measurements with an accuracy on the order of 0.01 g/cm^3^ [47].

Studies on nanoparticle‐based MRI CAs show how particle size, internal structure, and magnetization govern their relaxation efficiency. Experimental data across a broad range of maghemite particle sizes align well with theoretical predictions, enabling the construction of a master curve linking transverse relaxivity to particle dimensions. For individual magnetic crystals, only the crystal size and saturation magnetization are required to estimate T_2_ efficiency, while more complex assemblies, such as core‐shell systems, clustered cores, and vesicular structures, need an additional parameter describing the fraction of the total particle volume occupied by magnetic material. These relationships allow prediction of the maximum achievable relaxation effect and the particle dimensions required to reach it [48].

SPIONs are commonly utilized as T_2_‐negative CAs. However, conventional spherical particles often suffer from low crystallinity, relatively low transverse relaxivity, and the potential for false‐positive hypointense signals. A study reported a strategy to overcome these limitations by controlling nanoparticle morphology, showing that octapod iron oxide nanoparticles exhibit ultrahigh T_2_ relaxivity (679.3 ± 30 mM^−1^s^−1^) because of their effective magnetic core radius. These nanoparticles show improved performance compared to traditional spherical particles and enable for highly sensitive *in vivo* MRI for early tumor detection, especially in liver lesions [49].

## 6 Zeolites and Porous Materials for Bioimaging

This section focuses on zeolites and porous materials such as MOFs and mesoporous silica materials (MSMs) as emerging platforms for molecular imaging applications. It provides a general overview of zeolite structures in bioimaging, emphasizing their crystalline frameworks, tunable porosity, and high surface area, which enable the incorporation and controlled release of imaging agents. Specific zeolite topologies, including Mobil Five Structure (MFI), Linde Type L (LTL), Linde Type A (LTA), and Faujasite (FAU), are discussed in terms of their structural characteristics, pore architecture, and relevance to imaging performance. MOFs and MSMs are complementary classes of porous materials, with structural versatility, functionalization potential, and growing role in advanced molecular imaging and diagnostic applications.

### 6.1 Zeolite Structures

Zeolites represent a useful class of materials with great versatility, with potential uses in many research fields. Nowadays, with many concerns related to sustainability in environmental protection, its high capacity of cation exchange and good textural properties allows for the adsorption of greenhouse gases. The Scopus database gives a total of 49,543 works using an advanced search with terms “zeolite” and “environment remediation.” Moreover, other sectors linked to environmental issues are the chemical industry and petrochemical plants where they are used as catalysts, highlighting their role in energy‐related approaches such as biomass conversion and thermal energy storage as well [50, 51, 52].

The health‐related approaches involving zeolites as nanomaterials have been improving, with many outstanding properties. The Spansule formulation, introduced in 1952, was one of the first prolonged‐release drug technologies. It consists of a capsule filled with hundreds of tiny pellets, each coated with layers of a slow‐dissolving material of varying thicknesses. This coating controls how quickly the drug is released into the body, allowing for a sustained effect over several hours. Zeolites were introduced to give drug stability with versatility in its encapsulation and possibility for modification as a strategy to improve the drug delivery (DD) process [53, 54].

**FIGURE 4:**
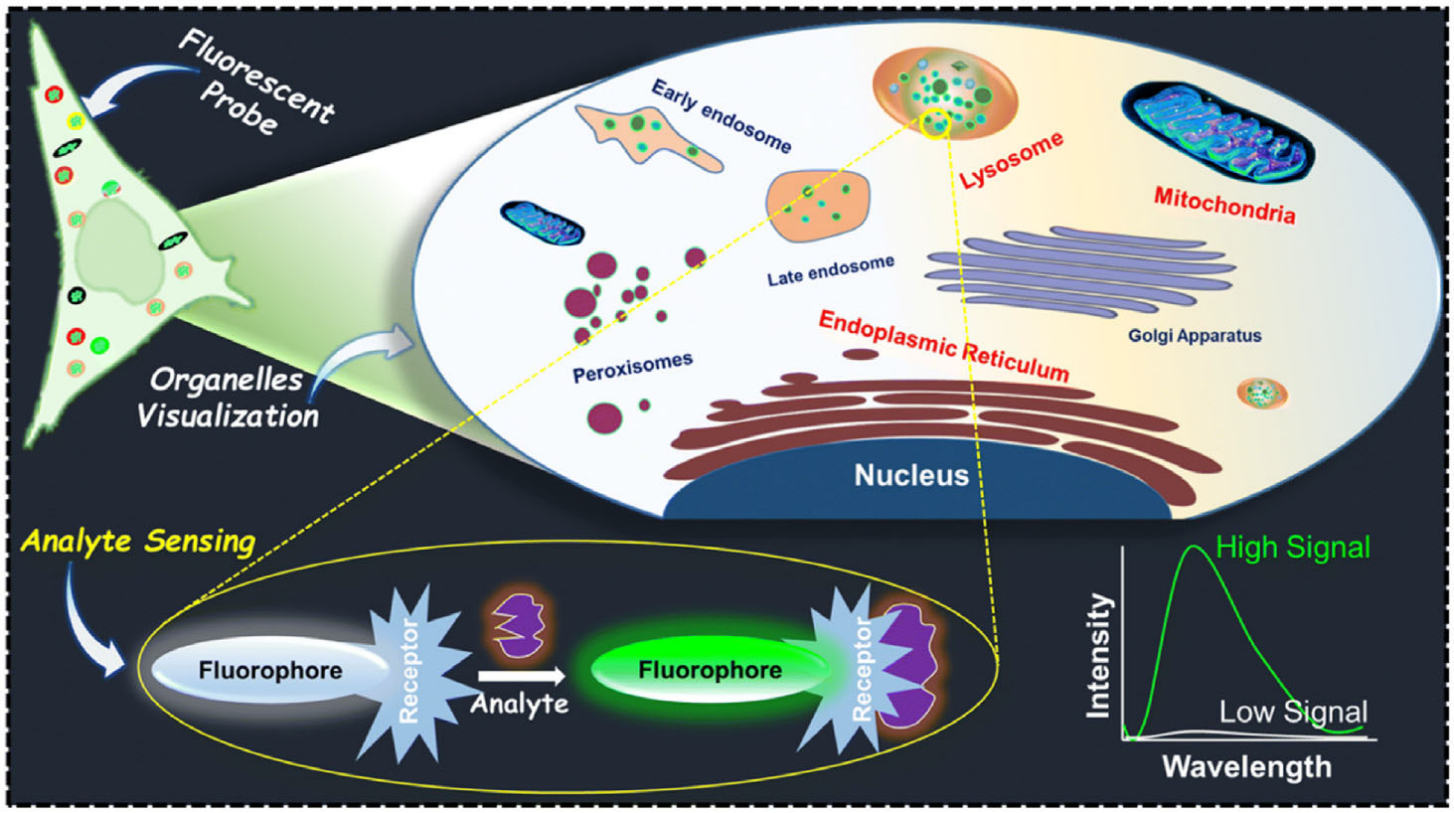
Illustration showing biosensing using a fluorescent probe. Reproduced with permission from Munan et al. [58]. Copyright 2024, Royal Society of Chemistry.

Other health related areas involving zeolites are in the biomedical field as CAs [55, 56, 57]. The development of bioimaging began in the 19th century with studies of fluorescence, showing the capacity of some organic molecules to absorb short wavelengths and re‐emit energy at longer wavelengths in the visible region. Sir George Stokes, an Irish scientist, developed the Stoke's law of fluorescence. Later on, the development of instruments such as spectrofluorometers and fluorescence microscopes allowed for detailed studies on the fluorescence phenomenon, for example for studying cellular processes, using small molecules and functionalization for constructing probes for imaging specific organelles [58]. Figure 4 shows the process, involving the detection of an analyte by the fluorescent probe, allowing for a better understanding.

The insertion of zeolites as a species of adjuvant for bioimaging is an inevitable process because of the use of nanotechnology for monitoring biological processes. This interdisciplinary field called nanobiotechnology, studies materials with biophysical properties that can be utilized for bioimaging, diagnosis, and contrast enhancement. It involves the integration of low‐cost related technology with fast detection of biomarkers from a single specimen [59].

Therefore, the integration of important features for biological applications of zeolitic materials such as biocompatibility, cellular viability, cellular uptake, and tissue response, as well as their morphological and textural characteristics make these materials, such as ZIF‐8 zeolites, attractive for use as drug delivery systems (DDS) and CAs [60, 61]. Other zeolitic topologies studied for carrying CAs include faujasite (FAU) in the form of GdNaY7, zeolite beta (BEA) [62], sodalite (SOD) in the form of a nanozeolite with Gd(III) [63], and NaA [56]. Therefore, zeolites are considered a theranostic platform, allowing for the union of therapeutic and diagnostic agents [64].

**FIGURE 5:**
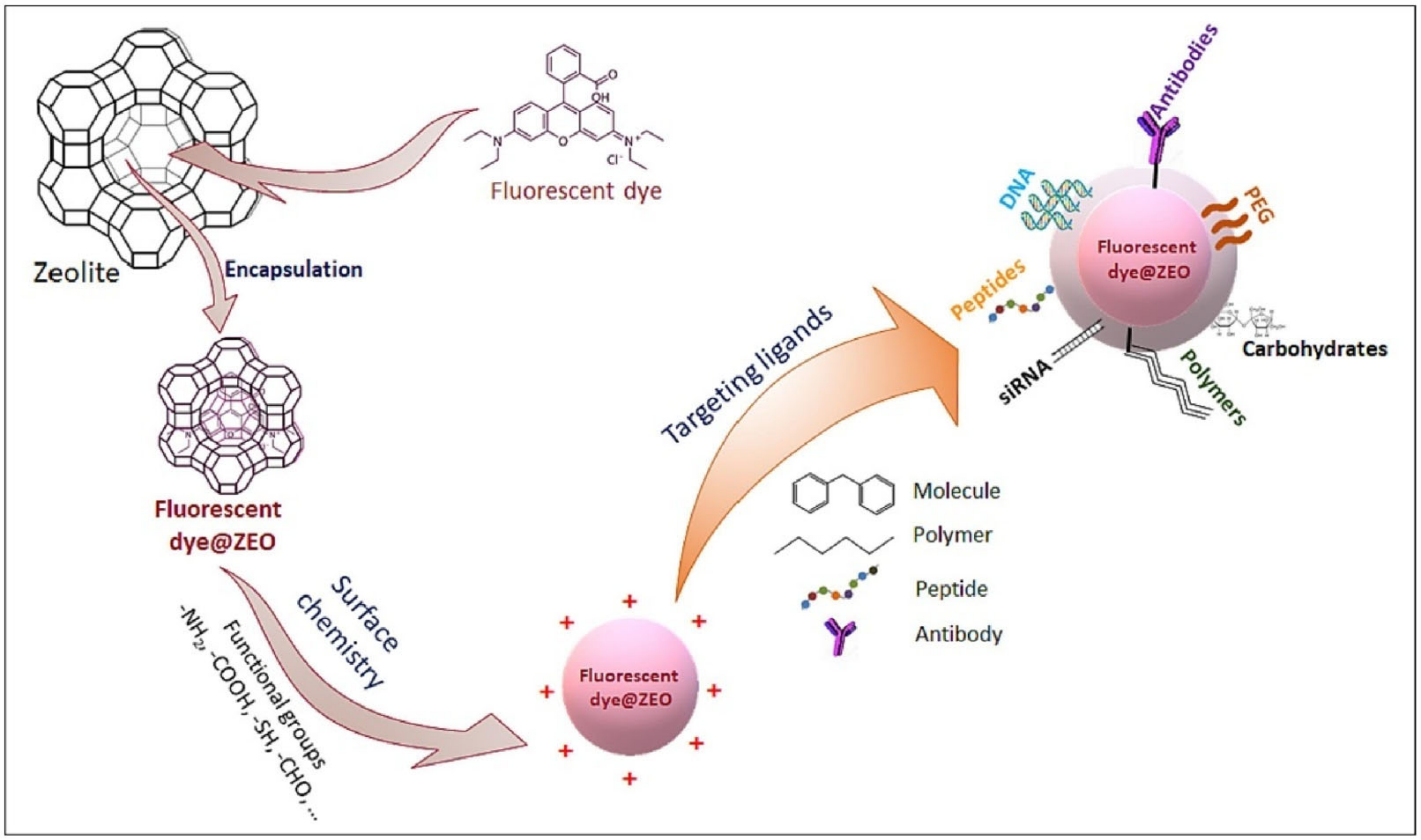
Zeolite structure modification with fluorescent dyes for use as probes for cancer cell bioimaging. Reproduced with permission from Bertão et al. [69]. Copyright 2023, Elsevier.

Since the development of medical techniques for diagnostics such as CT by Sir Godfrey Hounsfield in 1972, the use of nanomaterials has been important, helping as CAs to increase accuracy in locating many anatomical features and biological conditions such as broken bones, blood clots, internal hemorrhage, cardiovascular diseases, and tumors, allowing for precise local biopsy and surgery and monitoring after surgery [65]. Zeolites are an attractive alternative for treatment in oncology [66], with the ability to chemically modify their structures in order to be used for detecting tumor cells or exosomes, for cancer diagnostics [67]. The use of zeolites in MRI presents another approach where the chemical modification is an underlying aspect with exchangeable metal ions (Gd(III)), as was verified in the CA property of FAU zeolite nanocrystals, in monitoring occurrence of hypoxia in tumors [68]. Figure 5 shows zeolite structure modification with fluorescent dyes for use as probes for cancer cell bioimaging [69].

The following sections present the main characteristics studied of each representative zeolite topology with highlighted uses in bioimaging for diagnostics of the more reported conditions found in literature.

#### 6.1.1 Mobil Five‐Structure (MFI)

One of the modern methods used in medicine for cancer treatment is photodynamic therapy (PDT), where reactive oxygen species (ROS) are generated due to the interaction of photosensitizers with radiation. These ROS can be effective in destroying cancer cells. The photosensitizer is an organic molecule and the dye methylene blue has been used for loading for the zeolite ZSM‐5 (Zeolite Socony Mobil‐5) through adsorption. ZSM‐5 is one of the main representants of MFI topology that can be modified for better responses in PDT as it can be modified with PEG carrying methylene blue [70].

There are few examples of MFI zeolites for diagnosis using medical techniques. In this case, the capacity of these zeolites to transport photosensitizer molecules makes it possible to expand their use as bioimaging agents, mostly with ultrasound and PAI of cancer [71]. Zeolite‐catalase‐methylene blue nanocapsules based on hierarchical zeolites were used to treat pancreatic cancer, enabling ultrasound imaging‐driven oxygen self‐sufficient PDT [72]. The nanocapsules with 90% relative activity of equivalent free catalase enzyme efficiently modulated the tumor hypoxia and enhanced the intratumoral ultrasound contrast by sustained decomposition of endogenous H_2_O_2_ and in situ production of O_2_ gas bubbles. The methylene blue loading in hierarchical zeolite matrices prevented the rapid leaching of the photosensitizer in tumor tissue, achieving a good sustained photosensitizer release effect. Upon near‐infrared laser irradiation, the local cancer cells were completely killed, and no therapy‐induced toxicity and recurrence were observed.

#### 6.1.2 Linde Type L (LTL)

The nanosized material zeolite L or Linde Type L has been commonly used in biomedical applications, for example for diagnosis and for oxygen delivery in cancer treatment [73, 74]. The chemical formula of this zeolite is K_6_Na_3_(H_2_O)_21_[Al_9_Si_27_O_72_] with channels in three dimensions and doubled six‐ring (D6R) openings, resulting in 12 membered rings along the “c” axis. The high content of potassium makes possible the occurrence of ion exchange with gadolinium (Gd), mostly due to the extra framework K^+^ present where it can generate a system with high stability and unimodal distribution of particles for theranostic purposes [75].

The textural properties have direct relationship with the image generation when this zeolite is used. Paramagnetic Gd^3+^ ions can be inserted in large cages and Eu^3+^ ions introduced at small cages, forming a dual imaging probe due to the insertion and exclusion of water molecules in different regions of the body [76]. These lanthanide elements increase the MRI sensitivity and can influence the relaxivity rates of water proton spin states, even when the contrast agent is modified by biomolecules such as proteins [77, 78].

A paramagnetic complex of Gd called Gd‐DOTA (gadolinium‐tetraazacyclododecane tetraacetic acid) has been grafted in LTL zeolites [79], with the chelating of the contrast agent common to produce an increased signal related to the T_1_ relaxation time (longitudinal) [80]. The T_1_ and T_2_ (transverse) relaxation times are related to the brightness in images (e.g., of fat, edema, and cerebrospinal fluid). These times (T_1_ and T_2_) may be influenced by pH of the medium that zeolite Gd‐LTL structures are present in the body. It has been shown that at acidic pH values the T_1_ relaxivity decreases from 32 to 7 mM^−1^s^−1^ and the T_2_ relaxivity increases up to 98 mM^−1^s^−1^, indicating that the proton transfer, occurring from inside to outside of zeolites, influences the image generated at a level more relevant than water diffusion [74].

**FIGURE 6:**
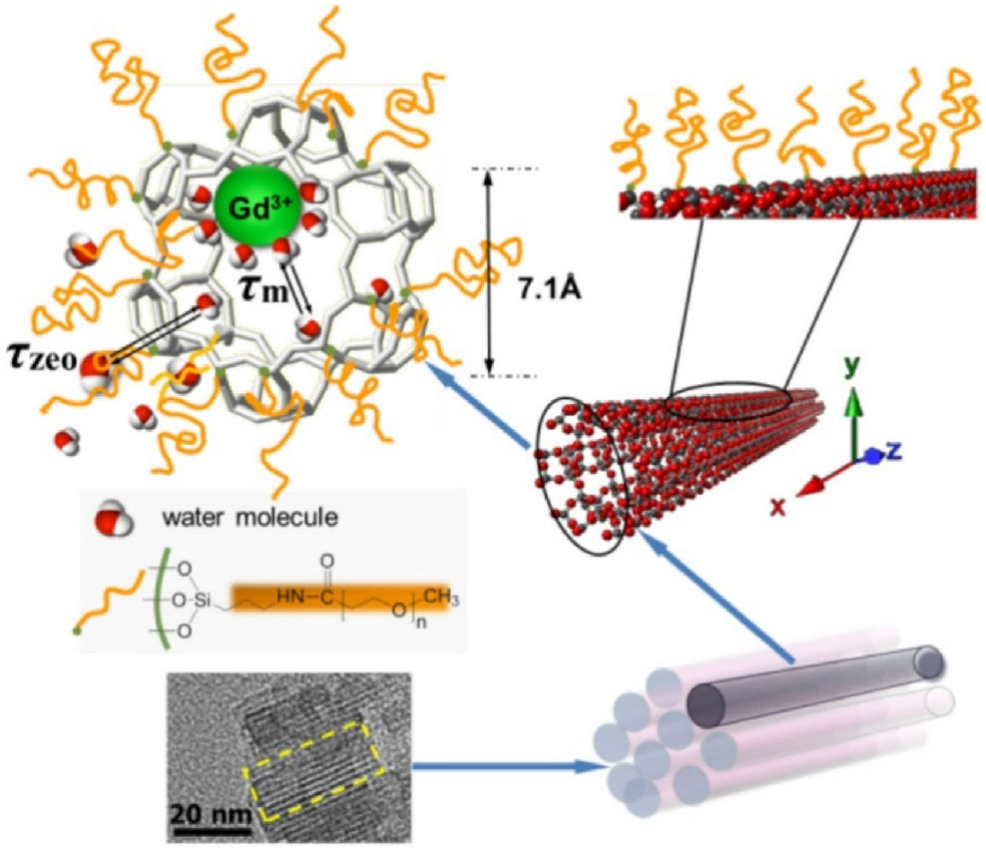
PEGylated Gd‐loaded LTL and the water exchange parameters: τ_m_ is the residence time of water molecules in the first coordination sphere of Gd(III) ions, and τ_zeo_ is the residence time of water molecules inside the zeolite. Only one cage unit is shown for convenience. Reproduced with permission from Zhang et al. [73]. Copyright 2017, ACS.

The modification of Gd‐LTL zeolites with a polymer such as PEG (i.e., *PE*Gylation) is a strategy for stabilization of the system, increasing the circulating time in vivo [81]. Figure 6 shows a PEGylated system with the times of residence of water molecules inside and outside.

#### 6.1.3 Linde Type A (LTA)

The Linde Type A zeolite, called NaA, is represented by the formula Na_12_(H_2_O)_278_[Al_12_Si_12_O_48_]_8_‐LTA and has been reportedly used as CAs in MRI [81]. The structure is considered to contain high amounts of aluminum and is suitable for insertion of radionuclides in the cages such as ^223^Ra, ^224^Ra and ^225^Ra, for radionuclide therapy [82]. Zeolite NaA can be labeled with ^223^Ra, specifically a radioimmunoconjugate formed with poly‐ethylene glycol, for therapy against metastatic castration‐resistant prostate cancer (mCRPC), showing high stability, cytotoxicity and therapeutic efficacy [83].

The versatility of NaA as a platform for carrying CAs is verified in its synthesis as nanocomposites with varied amounts of Fe_3_O_4_, a superparamagnetic component used for MR bioimaging. Table 2 shows the main characteristics of NaA/Fe_3_O_4_ obtained [56].

**TABLE 2:** NaA/Fe_3_O_4_ nanocomposite features for bioimaging using MRI.

**Nanocomposite** **name**	**%Fe**_**3**_ **O**_**4**_	Morphology	Magnetization saturation (emu/gFe)	**Susceptibility value** **(emu/gFe kOe)**	Magnetic resonance properties
IO_3.4_NaA	3.4	Cubic with average size 70–150 nm.	45.8	19.87	1/T_2_ relaxation rate increased with iron concentration; the T_2_ shortening effect was verified by the local magnetic field generated. The less the iron oxide content the better the image contrast.
IO_6.8_NaA	6.8		91.4	36.48	
IO_10.2_NaA	10.2		126.4	51.89	

The morphological characteristics of nano systems such as magnetic nanoparticles are important, as they influence relaxation rates for MRI. Relaxation rates can be quantified, helpful for measurements of tissue properties, not only for studies of anatomical abnormalities, but also for assistance in determining function and identifying loss of function in medicine [41, 84]. NaA zeolites have been used for diverse cancer therapies when incorporated with ^223^Ra, forming a stable system with high circulation time in the body and efficacy for treatment of glioma cancer cells [42, 44].

**FIGURE 7:**
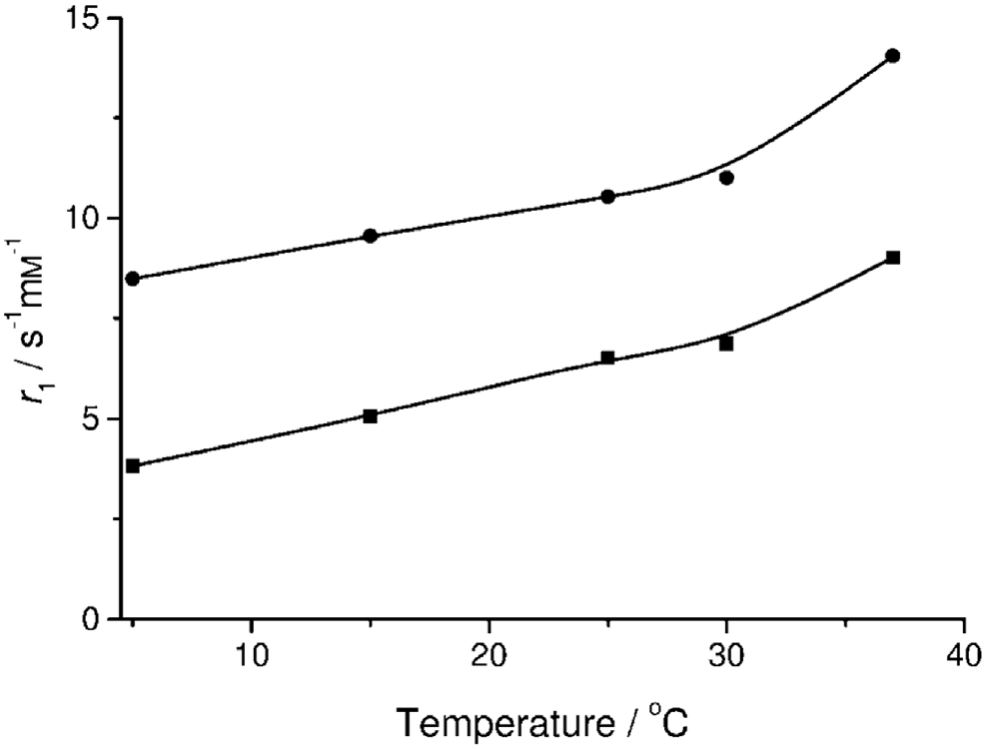
Temperature dependence of r_1_ relaxivities of GdNaA‐N‐1.5 (Si/Al = 1.0) (squares) and GdNaA‐M‐1.7 (Si/Al = 1.7) (circles) at 20 MHz. Reproduced with permission from Csajbók et al. [45]. Copyright 2005, Wiley‐VCH.

The incorporation of Gd^3+^ in NaA is dependent on its chemical composition, as it has been verified that the dealumination and incorporation of gadolinium has an effect on the relaxation time of water molecules inside the zeolite. This is due to the blocking effect of the channels, possessing a direct relationship with relaxation time, temperature, and water diffusion. Figure 7 shows the influence of temperature on the r_1_ relaxivities of different Gd‐NaA systems.

#### 6.1.4 Faujasites (FAU)

The faujasites are zeolite structures with wide uses as CAs in bioimaging and as therapeutic agents in DDS development. In accordance with the International Zeolite Association (IZA), the chemical formula of these structures is (Ca, Mg,Na_2_)_29_(H_2_O)_240_[Al_58_Si_134_O_384_]‐FAU. These nanomaterials are included in “theranostic” nanodevices and can deliver drugs within tumors, using the EPR effect [68]. Table 3 shows the different types of FAU zeolites, with their properties and ability to be modified and used for bioimaging.

**TABLE 3:** FAU zeolites for bioimaging in medical diagnostics.

FAU zeolite type	Modification	Imaging technique	Key features	Reference
NaY	Gd^3+^, Mn^2+^, and Ln^3+^ exchanged.	MRI	NaY zeolite as molecular imaging agents for oral administration in gastrointestinal tract and intravenous applications.	[57]
Not specified	Gd^3+^ exchanged for saturation with O_2_.	MRI	The textural properties of the nanosized zeolite allowed for effective gas transport for treatment of brain tumors. The FAU zeolite shortened T_1_ values, with a 2‐fold difference compared to Gd‐chelates.	[68]
Not specified	^64^Cu exchanged from [^64^Cu]‐Cl_2_.	PET	The loading of FAU zeolites with radionuclide ^64^Cu was achieved and quantified, for in vivo tracking in glioblastoma tumor cells in rats. Significant uptake of the zeolites was observed after 24 h with radioactivity stabilization for 48 h.	[85]
Not specified	Ag nanoclusters confined in microporous zeolites.	Luminescence imaging	The luminescence from the silver‐exchanged zeolite system was studied to determine its molecular sensing potential and use in light emission devices.	[86]
FAU‐X nanozeolite	Cu^2+^ and Fe^3+^ exchanged, with incorporation of Gd^3+^.	MRI	Nanosized zeolite was studied for theranostic applications, acting as hyperoxic/hypercapnic gas carriers for targeting tumor cells. The low content of iron and gadolinium allowed for effective visualization of tumors.	[87]
NaY	Modified with carbon dots.	Luminescence imaging	Various NaY zeolites modified with carbon dots were used as CAs in biomedicine.	[88]
NaY	Modified with carbon dots.	Luminescence imaging	Luminescence properties of the nanocomposite were investigated with phosphorescence and fluorescence bioimaging ability.	[89]
Not specified	Modified with quantum dots.	Luminescence imaging	Zeolites with quantum dots were investigated for their application in optical devices.	[90]

### 6.2 Metal‐Organic Frameworks (MOFs)

MOFs are materials produced by chemical bonding between organic ligands (e.g., cyanide, pyridyl, carboxylate, azolates, sulfonates, phosphonates, and hydroxyl) and metal ions such as Al^3+^, Cr^3+^, Fe^3+^, and Ti^4+^, forming crystalline structures with high porosity, functional diversity, and large surface areas [91, 92]. These materials exhibit a typically large internal surface area (500–10 000 m^2^/g), with low structural density (e.g., 0.124 g/cm^3^) and customizable pore sizes/characteristics (14–98 Å), allowing for the accommodation of diverse host structures [43, 93].

In general, MOFs are classified into three main types: 0D, 1D, and 3D. 0D MOFs exhibit discrete clusters or individual metal centers, while 1D MOFs exhibit chain‐like configurations. 3D MOFs develop extensive networks with porous architectures. MOFs can be broadly categorized into several classes, including isoreticular MOFs (IRMOFs), zeolitic imidazolate frameworks (ZIFs), and porous coordination polymers (PCPs), each offering distinct advantages [94].

Due to their high functionality, MOFs are widely used in biosensors, particularly for molecular detection and cell imaging, facilitating biomedical research and diagnosis. MOFs play a fundamental role in the development of biosensors for precise and selective detection. They are able to recognize specific molecules with greater accuracy. Furthermore, their low toxicity, high stability in water, good biodegradability, and biocompatibility are considered notable properties in the field [95].

There are two main approaches in MOF‐based bioimaging: (1) MOFs act as fluorescence quenchers for analyte fluorophores through mechanisms such as fluorescence resonance energy transfer (FRET), photoinduced electron transfer, or charge transfer, and (2) MOFs are designed with fluorescence or luminescence properties sensitive to their local environment or specific host molecules [96].

MOFs have a wide range of potential medical applications in bioimaging. CuS nanoparticles, known for their role in destroying cancer cells, absorb strong NIR light and are used as semiconductor optical agents. Using the Fe‐MOF system integrated with CuS nanoparticles, it was possible to synthesize a photothermal agent for imaging tumor cells. The CuS‐Fe‐MOF nanoparticles were fabricated by the codeposition/assembly method, in which a layer of Fe‐MOF was deposited on the surface of CuS nanosheets. When injected into mice with tumors, MRI and thermography allowed the localization of tumor cells [97].

Xiang et al. studied novel porous MOF‐derived Fe_3_O_4_@C (Fe_3_O_4_‐MOF) for their contrast‐enhancing effects in MRI. The results showed that the MRI signal intensity decreased with increasing concentration of Fe_3_O_4_ nanoparticles, with intensity values lower than that of conventional Fe_3_O_4_ nanoparticles. Furthermore, Fe_3_O_4_@C‐MOF nanoparticles, combined with alternating magnetic field (AMF) treatment, demonstrated effective therapeutic results in an oral cancer xenograft model. This system exhibited excellent MRI contrast, magnetic heating efficiency, and on‐demand drug release [98].

Nanoscale MOFs are commonly intrinsically radioactive for PET and serve as carriers of imaging and/or therapeutic cargos. Chen et al. produced and characterized UiO‐66 nMOF (^89^Zr‐UiO‐66), incorporating positron‐emitting isotope zirconium‐89 (^89^Zr) and carrying a peptide ligand (F3), targeting nucleolin in triple‐negative breast tumors [99]. The therapeutic doxorubicin (DOX) was loaded onto UiO‐66 with a relatively high loading capacity (1 mg DOX/mg UiO‐66), for therapy and visualization by fluorescence. Results revealed strong radiochemical and material stability in different biological media. Based on the findings from experiments, the nanostructures serve as an image‐guidable, tumor‐selective cargo delivery nanoplatform. In addition, toxicity evaluation confirmed that PEGylated UiO‐66 did not impose acute or chronic toxicity to test subjects. With selective targeting of nucleolin on both tumor vasculature and tumor cells, this nMOF can find broad application in cancer theranostics. Table 4 shows different MOF‐based compositions and their use for bioimaging in medical diagnostics.

**TABLE 4:** MOF‐based materials for bioimaging in medical diagnostics.

Composition	Imaging technique(s)	Key features	Reference
Zr‐based MOF	MRI, CT, and PET	Different MOFs with zirconium modification were used for drug delivery and bioimaging (e.g., in vivo tumor imaging).	[100]
2D‐Eu‐MOF (as biomarker); Mn(III)‐porphyrin MOF (bioimaging)	MRI, CT, PET, and SPECT	Modified MOFs can be used for gene detection and therapy and as fluorescent probes and CAs in multimodal imaging.	[101]
Cobalt ferrite and polydopamine MOFs	MRI	The ability of the system as a carrier for the anticancer drug gemcitabine was investigated as well as its potential for bioimaging.	[102]
ZIF‐90 with Zn^2+^ and imidazole‐2‐carboxaldehyde	A fluorescent nanoprobe encapsulating Rhodamine B (RhB) into ZIF‐90	The MOFs can be used for mitochondrial targeting with responsiveness towards adenosine triphosphate.	[103]
Fe‐MOF‐5‐NH_2_	MRI, fluorescence imaging (FI)	The system was used for drug delivery with appropriate monitoring by the imaging technique against HepG‐2 cancer cells.	[104]

In the early 2000s, NaY was significantly less developed and knowledge on the mechanisms of relaxivity and its relationship with drug release from these systems was limited. Later on, as zeolites and MOFs became more sophisticated, their use as CAs became realized [7]. Zeolites can act as carriers for specific agents and for imaging, with the nature of the CAs being ionic and commonly with metal oxides such as SPIOs.

It is important to highlight the importance and application of MRI as a technique in medicine, offering soft tissue contrast without the use of ionizing radiation, and providing good spatial resolution, with unlimited penetration depth [37]. The advent of multimodal imaging for diagnosis purposes emerged alongside advancements in MRI for cellular and molecular studies, due to better image resolution, with better sensitivity compared to other imaging techniques (e.g., CT) [105].

**FIGURE 8:**
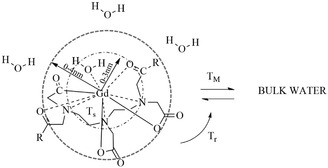
Gd^3+^ interaction with water molecules resulting in relaxation of water protons. Reproduced with permission from Xiao et al. [108]. Copyright 2016, Spandidos Publications.

Important parameters in MRI for bioimaging include the proton density, T_1_ relaxation time and the T_2_ relaxation time. These parameters heavily depend on the tissue properties and biological environment (e.g., medium) [106]. Due to their properties, zeolites and MOFs are attracted by magnetic fields due to unpaired electrons. The protons of water molecules are excited to a higher energy level, with the paramagnetic metals encountering these protons, returning the molecules to the lower energy level. The concentration of the CA is important as it affects the T_1_ (longitudinal) and T_2_ (transverse) relaxation times. In the relaxation process there is a loss of energy to realign the nucleus due to the magnetic field, while in the dephasing there is a loss of energy due to the loss of precessional phase. The times for these two phenomena are T_1_ and T_2_ respectively, with the image being generated due to these processes and the energy given off [107, 108, 109, 110]. Figure 8 shows the interaction of a CA with water molecules.

### 6.3 Mesoporous Silica Materials (MSMs)

Since their discovery in the 1990s, mesoporous materials have been used in a wide variety of technological fields, especially in materials science, environmental science, food science, and healthcare. Silica‐based materials classified as mesoporous are those that have an average pore diameter in the 2–50 nm range, giving these materials structural properties that are highly valued for use in biomedical applications.

MSMs are classified into different families/classes, based on their structural differences and methods used to construct them. The classes of MSMs include the Mobil Composition of Matter (MCM), Santa Barbara Amorphous materials (SBA‐*n*), Fudan University ordered mesoporous materials (FDU‐*n*), Korea Institute of Technology mesoporous materials (KIT‐*n*), and periodic mesoporous organosilicas (PMOs) [111, 112, 113, 114, 115, 116, 117, 118, 119]. These MSMs have diverse applicability due to their advanced multifunctional properties and adaptability to different microenvironments.

MSMs are commonly prepared via the conventional sol‐gel or hydrothermal methods. The hydrothermal method requires the use of a stainless‐steel reactor, which considerably reduces the synthesis time of the nanostructures, since it is possible to control the nucleation and growth of mesoporous nanoarchitecture crystallites in a microenvironment under controlled pressure. It is very common to use a template as a structure‐directing agent and a silica source. The type of template used is highly dependent on the type of mesoporous arrangement required, but the most commonly used are hexadecyltrimethylammonium bromide (C_16_TAB or CTAB), trimethyloctadecylammonium bromide (C_18_TAB), poly(ethylene glycol)‐*block*‐poly(propylene glycol)‐*block*‐poly(ethylene glycol) (Pluronic P123), and poly(ethylene oxide)‐*block*‐poly(propylene oxide)‐*block*‐poly(ethylene oxide) (Pluronic F‐127), as well as, some ionic liquids. The silica sources, which are responsible for the formation of the amorphous silica framework, for the production of MSMs, include tetraethyl orthosilicate (TEOS), tetramethyl orthosilicate (TMOS), sodium silicate, and fumed silica (e.g., Aerosil), as well as some alternative silica sources, such as rice husk ash, sugarcane ash, and corn straw ash [120, 121].

The use of MSMs in the biomedical field is possible due to the unique features of these nanomaterials, from their unique textural and structural characteristics. These characteristics include (i) the high density of silanol groups, which are responsible for anchoring different functional groups; (ii) the well‐defined and rigid mesoporous walls and channels, which guarantee high efficiency of diffusional processes in biological media; (iii) the high content of amorphous silica, allowing the mesoporous array to be nontoxic in the biological microenvironment; (iv) the high surface area, which allows the incorporation/loading of different drugs and active agents within the mesoporous matrix; (v) the adaptable and adjustable mesopores, according to the size of the drug for controlled release applications; (vi) the thermal and mechanical stability, allowing for easy diffusion of active agents in biological media, as well as acting as a protective cage for therapeutic agents (e.g., drugs) against thermal and chemical damage [120, 122, 123, 124, 125].

MSMs are extremely useful and efficient for use in controlled drug delivery systems, cancer treatment, gene delivery, antibacterial therapies, tissue engineering and regenerative medicine, biomedical imaging and theranostics, PDT and photothermal therapy (PTT), biosensors and biomarkers, disease diagnostics and monitoring, and in the development of new CAs.

MSMs have been used in various applications in the biomedical field. Vale et al. prepared a hybrid mesoporous array (i.e., MCM‐48/FeNPs) for use as a PAI contrast agent [126]. PAI is an attractive technique in the biomedical field because it does not require the use of ionizing radiation and provides high‐resolution images. The mesoporous material MCM‐48 was prepared via the sol‐gel method, while the mesoporous hybrid array MCM‐48/FeNPs was obtained from the post‐functionalization methodology. MSMs presented spherical particles with an average size ranging between 250 and 270 nm, with a highly ordered 3D mesopore arrangement, as well as high surface area values, which were 779.2 and 171.7 m^2^/g for the MCM‐48 and MCM‐48/FeNPs structures, respectively. It was observed that the hybrid mesoporous material MCM‐48/FeNPs proved to be efficient as an effective photoacoustic contrast agent, since the mesoporous matrix modified with FeNPs significantly contributed to the amplification of the signal generated by the PAI technique.

Salari‐Goharizi et al. reported the synthesis of the mesoporous silica matrix SBA‐15, which has textural and structural properties that make it a strong candidate for use as a contrast agent for diagnosis and imaging of diseases, as well as for use as a nanocarrier for DOX release [127]. SBA‐15‐based mesoporous array was synthesized via the hydrothermal method using TEOS and Pluronic P123 as silica source and structure‐directing agent, respectively. SBA‐15‐based array exhibited the characteristic X‐ray diffraction peaks of the (100), (110), and (200) diffraction planes typical of a hexagonal phase mesostructure with type IV isotherm, a high surface area value (884 m^2^/g), as well as a distribution of mesopores within the range of mesoporous materials with high structural order and high thermal and mechanical stability. The mesoporous hybrid based on SBA‐15 showed excellent loading efficiency and controlled release of DOX, which has proven effective for use in targeted administration and controlled release in cancer cells, as well as in cancer‐targeted imaging technologies.

Khan et al. used mesoporous silica nanoparticles modified with Gallium‐68 (^68^Ga) (^68^Ga‐MSNPs) as CAs in single‐cell PET imaging [128]. The production of the ^68^Ga‐MSNPs‐functionalized mesoporous silica was achieved by modification of pure MSNPs with ^68^Ga using a chelator‐free method. The maximum radiolabeling capacity of ^68^Ga into the mesoporous silica was greater than 30 GBq/mg. The fractions of ^68^Ga adsorbed by 0.1, 1, 10, and 100 µg of MSNPs were approximately 4.3%, 6.9%, 43.3%, and 83.5%, respectively, corresponding to loading efficiencies of 25.8, 4.14, 2.60, and 0.5 GBq/mg. The uniform and thermodynamically stable ^68^Ga‐MSNPs also showed good cellular biocompatibility.

Li et al. prepared a silica‐based mesoporous array modified with Prussian blue for cancer treatment using PTT, as well as for enhancement of ultrasound imaging [129]. The Prussian blue and the mesoporous array PB@mSiO_2_ were synthetized using hydrothermal and sol‐gel methods. The authors also evaluated the incorporation and controlled release of perfluorohexane (PFH) within the mesopores of the architecture. The incorporation was performed via a vacuum infusion process to form the PB@mSiO_2_‐PFH matrix. The release of PFH was evaluated in phosphate‐buffered saline (PBS), while the ultrasound contrast of PB@mSiO_2_‐PFH was measured in tumor‐bearing mice. The particles of the mesoporous array exhibited a cubic shape with an average particle size of 207.1 nm, high surface area and high pore volume. The results show that the mesoporous material exhibited excellent hemocompatibility, low toxicity, and high thermal sensitivity towards tumor cells. Furthermore, the maximum PFH loading rate in the mesopores of the nanoarchitecture array was around 30.73% by weight. The functionalized mesoporous matrix PB@mSiO_2_‐PFH exhibited remarkable photothermal effects and improved ultrasound image contrast from the use of laser irradiation at 808 nm.

Mazhit et al. prepared a mesoporous material based on barium (Ba)‐modified silica (i.e., SiO_2_‐Ba NPs), for use as a contrast agent in CT [130]. The synthesis of the mesoporous matrix was carried out using the sol‐gel method, in which TEOS was used as a silica source, CTAB as a structure‐directing agent, and sodium hydroxide (NaOH) as a catalyst. In addition, the post‐functionalization of the mesostructure with Ba was performed using barium nitrate (Ba(NO_3_)_2_) as a structure‐modifying agent. Considering its textural and structural characteristics, the functionalized mesoporous matrix presented a type IV isotherm, according to the IUPAC classification, with a particle size ranging from 15 to 45 nm, and interconnected mesopores in a silica framework with a high surface area. The Hounsfield unit (HU) values of the mesoporous matrix were higher than those of iopromide, whose values were ∼323.6 ± 4.7 HU/g·L and ∼70.7 ± 2.2 HU/g·L, respectively, thus indicating that the Ba‐functionalized mesoporous matrix is superior compared to conventional CT imaging agents. Furthermore, the functionalized mesoporous array exhibited an X‐ray attenuation efficiency approximately 4.5 times greater than the conventional iodine‐based molecular contrast agent iopromide.

Huang et al. prepared a series of blue fluorescent magnetic PMOs, functioning as nanocarriers for gambogic acid, for targeted delivery in VX2 tumor cells through SPECT image guidance [117]. The double template approach followed by the post‐functionalization method via carbon quantum dot grafting were used to prepare the fluorescent magnetic array PMOs‐HS@CDs and magnetic material PMOs‐MHS@CDs. The prepared PMOs presented a cubic crystal system with particle size between 150 and 200 nm, a framework composed of multiple layers in a well‐ordered mesoporous external architecture, high surface area, and low saturation magnetization. The prepared PMOs‐HS@CDs and PMOs‐MHS@CDs showed gambogic acid loading efficiencies of approximately 92.16%, 92.86%, 91.86%, 85.63%, 84.75%, and 84.13% and 88.85%, 87.39%, 87.30%, 84.42%, 79.96%, and 79.38%, for 1, 2, 3, 5, 7.5, and 10 mg of gambogic acid, respectively. In addition, the PMOs‐HS@CDs‐GA showed a release efficiency of 29.56% (pH 5.7) and 16.71% (pH 7.4) of the gambogic acid after 86 h. Finally, cytotoxicity studies revealed that all PMOs are virtually nontoxic and useful as drug nanocarriers in biomedicine, as well as for targeted SPECT imaging, showing greater ^99m^Tc uptake using PMOs‐MHS@CDs in tumor areas, compared to untargeted tumor areas.

Wu et al. prepared a rod‐shaped hollow mesoporous silica system as a nanocarrier for ibuprofen, as well as for optical imaging [131]. The mesoporous silicas were prepared using TEOS as a silica source. The hollow mesoporous silica nanorods (HMSNR) were prepared via the cocondensation method using Gd(OH)_3_ as a template, while the HMSNR@Gd_2_O_3_:Eu material was prepared using GdCl_3_ and EuCl_3_. Analysis of ibuprofen in the luminescent mesoporous material HMSNR@Gd_2_O_3_:Eu revealed an incorporation rate of 19.4%. Ibuprofen release occurred around 12 h, resulting in a release efficiency of 44.9%, as well as a cumulative release of 80% after 72 h. Furthermore, the tests performed on NCI‐H460 lung cancer cells indicate that the prepared mesoporous matrix HMSNR@Gd_2_O_3_:Eu shows great promise for use in diagnostics.

Dai et al. prepared multifunctional hollow mesoporous silica nanospheres for controlled drug delivery and MRI in cancer treatment [111]. The mesoporous silicas were prepared via a modified Stöber method, followed by the post‐functionalization method of the initially prepared silica particles. Furthermore, studies involving biocompatibility, cellular uptake, cytotoxicity, apoptosis, and in vivo imaging were performed using the 4T1 mouse breast cancer cell line, while controlled release tests were carried out by incorporating paclitaxel into the mesoporous matrix. The tests revealed that the mesoporous matrix exhibited a paclitaxel loading capacity of approximately 77.38 μg/mg, with the MRI signals increasing significantly due to the inclusion of the mesoporous silica matrix in the microenvironment.

Chen et al. developed a metformin nanocarrier based on mesoporous silica using TEOS as a silica source and employing a novel in situ synthesis method [113]. The controlled release of metformin reached a cumulative release of 67.1% (pH 6.5, tenth day). The photothermal conversion efficiency of the silica‐based nanostructures were around 42.7% and 59.9%, for the CuS@MSN and CuS@MSN@PDA nanoarchitectures, respectively. Furthermore, the use of nanomaterials led to a 80% lower tumor recurrence rate compared to the metformin only group.

## 7 Metallic Nanoparticles for Diagnostic Imaging

Contrast agents play a key role in CT, enabling the diagnosis and monitoring of small lesions and more complex diseases. In general, the most commonly used CAs are organic iodine‐based compounds (atomic number “Z” = 53) with three iodine atoms per molecule or six per dimeric molecule, but they have limitations related to short blood circulation times, the need for high injection doses, and nonspecific accumulation in intra/extravascular regions, which can cause renal toxicity, with various side effects [1, 31, 132].

MNPs are useful for various applications due to their high surface‐to‐volume ratio, as well as their optical and antibacterial properties. Another factor is that surface modifications can achieve desirable biocompatibility and colloidal stability [133, 134, 135]. In the biomedical field, these nanostructures can be applied in cancer therapy, drug delivery, and X‐ray attenuation [136]. MNPs are considered promising CAs in CT because they have a high density of metal atoms, high X‐ray attenuation coefficients (*η*), longer blood circulation times, and can integrate molecules on their surface for targeting specific diseases, organs, and tissues [137, 138]. In recent years, X‐ray fluorescence (XRF) imaging has gained attention as it is a highly sensitive molecular imaging technique that can detect characteristic X‐ray emissions from heavy metal nanoparticles (e.g., gold, molybdenum) in biological tissues. It is often used with CT imaging for simultaneous functional and anatomical mapping in vivo, with submillimeter resolution, elemental specificity, and high penetration depth. It is an important technique for quantitative and comparative assessments in biodistribution and pharmacokinetic studies of nanomedicines [139, 140, 141, 142].

In the study by Kim et al., PEG‐coated AuNPs measuring approximately 30 nm were tested as CAs, proving to be up to 1.9 times more efficient than the commercially available agent Ultravist [143]. In addition, the nanostructures had a longer blood circulation time (>4 h) than the commercial product (10 min), and CT images of the treated animals showed clear delineation of the cardiac ventricles and large vessels. Furthermore, after intravenous injection of AuNPs‐PEG, animals with hepatocellular cancer showed improved contrast in relation to normal liver tissue.

Sun et al. produced AuNPs coated with heparin and the amino acid 3,4‐dihydroxyphenylalanine (DOPA) measuring ∼55 nm in size and used them as imaging agents in CT, specifically for the liver [144]. Two hours after injection, the contrast efficiency generated by the AuNPs was 21.9 HU/mM in the liver, while the commercial iodinated contrast agent eXIA 160 showed a contrast efficiency of 4.2 HU/mM. Contrast from NPs remained for up to 24 h, showing that nanostructures can be used as molecular probes and imaging agents in the monitoring of liver cancer.

Rand et al. developed an imaging technique for the early diagnosis of hepatocellular cancer using AuNPs [145]. Tissues labeled with 10 or 50 nm electrodense nanostructures exhibited greater X‐ray scattering compared to normal tissues, differentiating cells containing AuNPs from those without the nanomaterial. This approach facilitates the in vivo detection of tumors as small as a few millimeters in size.

AuNPs have been reported in previous studies with applications in other types of pathologies, such as in the study by Hainfeld et al., in which 1.9 nm AuNPs were injected intravenously into mice and CT images were recorded for longer periods, due to the slower elimination of nanostructures from the blood compared to iodine‐based agents [146]. The results showed that organs and tissues such as kidneys and breast tumors were visualized more clearly and with high resolution. In addition, blood vessels less than 100 µm in diameter could be outlined, enabling vascular modeling. Histology of organs determined no evidence of toxicity for up to 30 days after injection.

Popovtzer et al. developed a targeted imaging platform based on AuNPs that enabled the detection of head and neck cancer at the cellular and molecular levels [18]. Inducing distinct contrast in CT images, the particles selectively bind to specific tumor antigens, with X‐ray attenuation over five times higher when compared to untargeted cancer cells or noncancerous cells.

**FIGURE 9:**
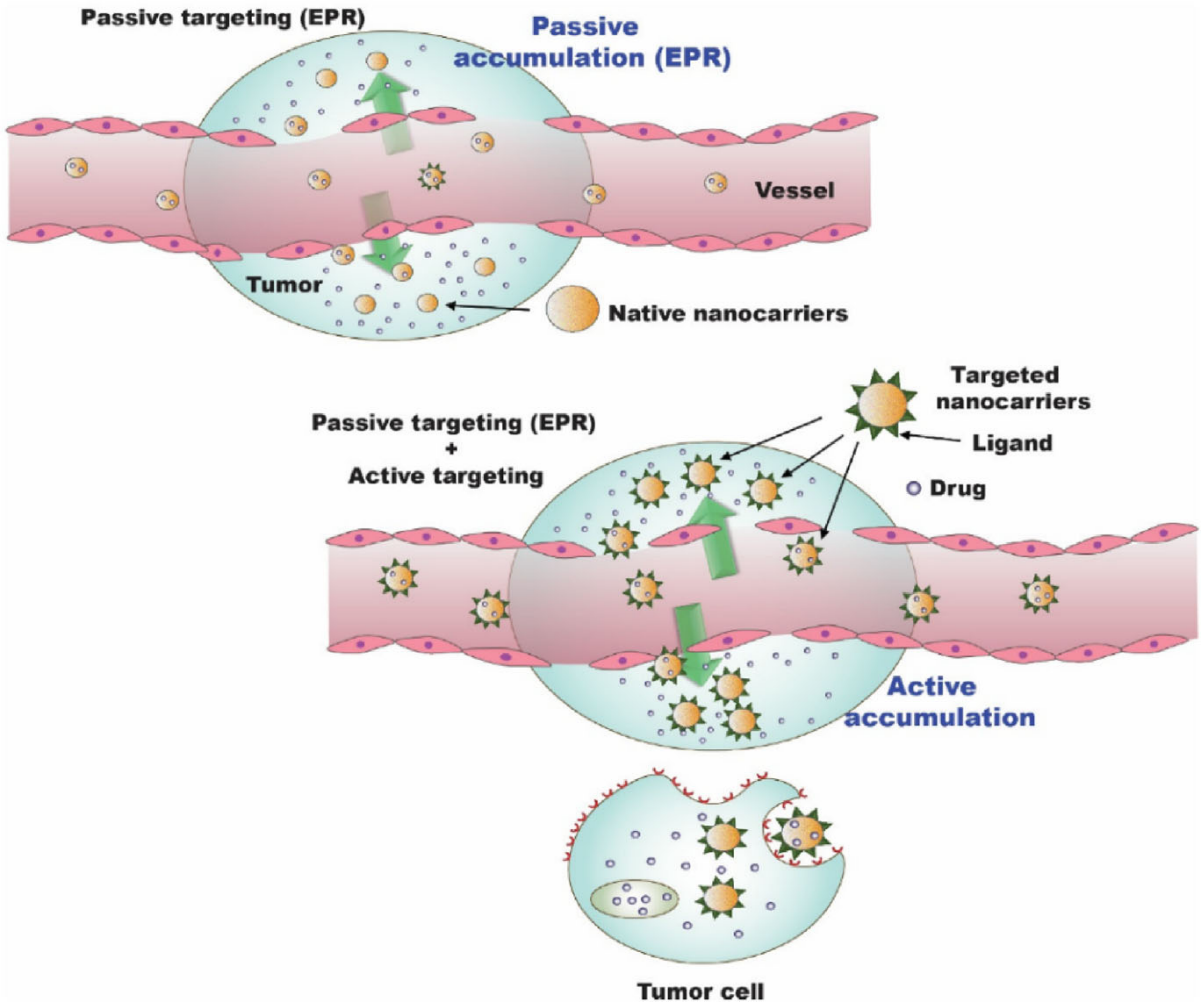
Main targeting approaches in cancer therapy. Reproduced with permission from Sarfraz et al. [147]. Copyright 2023, MDPI.

Reuveni et al. synthesized 30 nm AuNPs conjugated with anti‐EGFR antibodies and, after intravenous injection in mice, observed skin tumors in CT images, demonstrating more efficient and specific active targeting of the tumor region (Figure 9) [19]. This molecular cancer imaging tool is noninvasive and can facilitate early detection of the disease, in addition to allowing in vivo investigation of the expression and activity of biomarkers in molecular processes for various pathologies.

Cui et al. produced BSA‐stabilized silver nanoparticles (AgNPs) of approximately 5.8 nm, administering them intratumorally, for improving contrast at the site of the breast tumor in mice [20]. Over time, the signal from nanostructures gradually decreased in the bladder and tumor, proving that AgNPs‐BSA were cleared by the kidneys. Using PTT, the nanoscale particles were effective after intratumoral injection, causing cell death.

Lyu et al. synthesized bimetallic iron‐palladium (FePd) nanoparticles using NaBH_4_ as a reducing agent and then coated them with cysteamine (Cys) to improve colloidal stability and biocompatibility [21]. The results show spherical particles with an average diameter of 3.4 nm that, when applied in biological assays can be used as trimodal CAs, having greater X‐ray attenuation efficiency (2.6 HU/mM) compared to commercial contrast media such as iopromide (2.0 HU/mM). In addition to improving CT signals and satisfactory contrast in vivo, the NPs can be used for MRI imaging of breast cancer. The nanostructures showed a synergistic effect in PTT and radiotherapy in vitro, since, after thermoradiotherapy, they induced significant tumor ablation.

Surface coating is also a promising strategy for synthesizing MNPs for contrast imaging. In this regard, Wang et al. produced platinum nanoparticles (PtNPs) and applied them in in vivo experiments for angiography, with signals gradually decreasing over 4 h before stabilizing at normal serum levels [22]. Surface modification with bovine serum albumin (BSA) provided greater X‐ray attenuation (16.8 HU/mM), with prolonged imaging time, important for clinical applications.

In the study by Tang et al., PtNPs of 6.7 nm size were synthesized by chemical reduction with NaBH_4_, with coordinated assembly using human serum albumin (HSA) [148]. Higher X‐ray attenuation efficiency (5.6 HU/mM) was achieved compared to the contrast medium iopromide (5.2 HU/mM). Additionally, after intratumoral injection at a dose of 250 µmol Pt/kg, greater contrast was observed using CT at the breast tumor site compared to the results of the group treated with iopromide. This is attributed to the greater tumor retention of the nanostructures, which can be used for potent hyperthermia and photothermal ablation of tumors.

Zhang et al. synthesized highly stable (−35.5 mV) and monodisperse (13.5 nm) hyaluronic acid‐coated AgNPs that exhibited low cytotoxicity against macrophages in vitro [149]. The nanostructures had strong X‐ray attenuation (3.5 HU/mM) dependent on the Ag concentration, making them excellent candidates for applications as highly sensitive CAs. Along with CT, the NPs can be used for SPECT imaging, demonstrating efficient accumulation in tumors.

Silver sulfide (Ag_2_S) nanoparticles of 3.1 nm size conjugated with glutathione were produced by Hsu et al. and investigated for their potential as CAs in vivo using CT [150]. The results showed that, after intravenous injection (250 mg Ag/kg), contrast enhancement was observed in the bladder, heart, and kidneys, with at least 85% of the nanostructures being excreted in the urine within 24 h. Low accumulation was observed in the liver and spleen, with results suggesting that the Ag_2_S NPs are promising agents for clinical applications.

The diameter of nanoscale particles can strongly influence contrast observed using CT, as demonstrated in the study by Tsvirkun et al., in which GB111‐PEG‐coated AuNPs labeled with cathepsin inhibitor showed X‐ray attenuation efficiency between 22 and 25.2 HU/mM, while the iodinated contrast agent used had attenuation efficiency between 4 and 5.4 HU/mM [151]. This nanotechnology‐based platform is promising for functional imaging of specific protease activity. In vivo experiments after intravenous injection demonstrated that CT signals at the breast tumor site were higher when using nanostructures up to 30 nm compared to 100 nm particles.

Dong et al. synthesized AuNPs‐PEG with varying sizes and found that smaller nanostructures (4 and 15 nm) provided greater CT contrast for longer periods because the mononuclear phagocytic system had more difficulty clearing them, resulting in higher blood circulation times [152]. For AuNPs above 50 and up to 152 nm, this accumulation occurred in the liver and spleen, where contrast images were better.

In another study, Ce6‐PEG‐coated PtNPs with a diameter of around 70 nm improved CT contrast at the breast tumor site after intravenous injection of 100 μL NPs (1 mg/mL) in BALB/c mice. These NPs have the potential to be used for PAI, thus being an important tool for monitoring the hypoxic tumor microenvironment [153]. In addition, biochemical assays showed no evident toxicity, as results from serum biochemistry did not differ significantly from healthy animals, and histology found no damage to tissues of the organs of mice treated with the nanostructures.

Other studies highlight small MNPs as effective CAs, as is the case with the results described by Chou et al., in which biocompatible iron‐platinum (FePt) nanoparticles with diameters of 3, 6, and 12 nm showed potential for CT and MRI [154]. In addition, the nanostructures had excellent biocompatibility (cell viability >75% at 0.1 M) and hemocompatibility (<5%) in all experiments, also allowing conjugation with monoclonal antibodies and other functional ligands. Particle size influenced their properties, as the use of 12 nm FePt nanoparticles resulted in the highest serum concentration, circulation half‐life, and X‐ray contrast, while 3 nm particles showed the highest concentration in the brain, possibly ideal for brain mapping.

More recently, García et al. synthesized albumin‐stabilized cerium oxide nanoparticles (CeO_2_ NPs) with a diameter of around 5 nm that were administered to mice bearing xenografted subcutaneous sarcoma tumors [155]. The results revealed a considerable increase in X‐ray contrast, with the use of concentrations up to 10 times lower than commercial CAs. After injection into the caudal vein, CeO_2_ NPs exhibited absorption of around 85% of the injected dose by the liver and spleen, while in the tumor this absorption was around 99% and remained for up to 7 days, with no accumulation detected in the blood vessels, indicating good solubility, dispersion, and observation of the disease.

For contrast imaging, MNPs are directed to the cell surface where they can adhere and recognize specific receptors or biomarkers expressed by the affected cells. After facilitating this interaction, the targeting ligands initiate internalization processes, and MNPs, which have favorable physicochemical properties and produce detectable signals, can move throughout the cell, thereby tending to increase the contrast observed using imaging modalities, such as CT, MRI, and optical imaging [65, 156].

Applications in the field of theranostics, which involve nanoparticulate agents acting as dual therapeutic drug carriers and imaging CAs, can be considered revolutionary as they pave the way for simultaneous diagnosis and treatment, providing individualized and integrative approaches to health, greatly advancing the medical and imaging research fields [157, 158, 159]. In recent years, the development of improved nanostructure designs for the advancement of personalized medicine has come to the fore, in addition to nanoparticle‐mediated drug delivery and molecular‐level imaging. Together, this will enable early detection, accurate diagnosis, and treatment approaches with clinical trials at different stages aimed at regulatory approvals for the use of nanoscale particles and the assistance of artificial intelligence (AI) in the field of biomedical imaging.

## 8 Magnetic Nanoparticles for MRI

Nanotechnology has emerged as a transformative field in the 21st century, driving the development of advanced analytical and synthetic methodologies that enable the manipulation of matter at the nanoscale with unprecedented precision. This technological evolution has been particularly impactful in biomedical imaging, where engineered nanomaterials have allowed the creation of CAs exhibiting improved sensitivity and biocompatibility [160, 161]. Over the past decade, the integration of nanotechnology with multimodal imaging platforms—ranging from traditional techniques such as CT to advanced modalities including PET, SPECT, ultrasound, and MRI—has profoundly reshaped diagnostic strategies in clinical and research settings [162, 163].

MRI stands out as a noninvasive imaging modality and offers exceptional soft‐tissue contrast, enabling the detailed visualization of anatomical and physiological alterations in a wide range of pathological conditions, such as brain lesions, cancer, inflammatory diseases, muscular degeneration, and cerebrovascular disorders [3]. Human tissues contain 70–90% water, and pathological regions frequently display altered water content. Upon exposure to a static magnetic field, typically 0.5–3 T in clinical MRI and up to 10.5 T in advanced research MRI, the magnetic moments of water protons align with the external field. Radiofrequency (RF) pulses excite these protons, causing them to move to higher energy states. When the excitation ceases, the protons relax to equilibrium, generating signals governed by longitudinal (T_1_) and transverse (T_2_/T_2_*) relaxation times, which constitute the basis for MRI image contrast [164, 165].

Despite its inherent diagnostic power, endogenous contrast in MRI is often insufficient to discriminate between subtle physiological changes. Thus, CAs play a central role in enhancing resolution, sensitivity, and tissue specificity. Conventional small‐molecule gadolinium chelates remain widely used. However, concerns about NSF, brain deposition, and off‐target accumulation have stimulated intense efforts to develop safer and more efficient alternatives [41, 42]. In this regard, magnetic nanoparticles—including iron oxide nanoparticles (IONPs), ultrasmall iron oxide nanoparticles (USPIONs), manganese‐based nanostructures, and hybrid ferrite systems—have emerged as state‐of‐the‐art candidates for T_1_‐, T_2_‐, and dual‐mode T_1_–T_2_ contrast enhancement [46].

Recent developments between 2022 and 2025 have significantly expanded the magnetic nanoparticle landscape. Advances in controlling crystallinity, surface engineering, hydrophilic coatings, and hybrid core–shell structures have resulted in nanomaterials with enhanced magnetic susceptibility, improved relaxivity, longer blood circulation times, and reduced mononuclear phagocyte system (MPS) uptake (Table 5). These properties allow for more efficient modulation of proton relaxation, offering greater contrast enhancement and expanding the scope of MRI applications—from high‐field morphological imaging to emerging low‐field MRI systems, theranostic integration, and precision oncology.

**TABLE 5:** Magnetic properties and blood circulation times of some metal‐based nanoparticles used for bioimaging.

Type of magnetic NPs	**Relaxivity values, mM** ^**−1**^ **s** ^**−1**^	Circulation times	References
Manganese‐based	r_1_ = 0.15 (Mn_3_O_4_, 3 T), 5.99 (MnO, 1.5 T), 29 (MnO_2_, 1.5 T) r_2_ = 3.2 (Mn_2_O_3_, 1.41 T), 5 (Mn_3_O_4_, 3 T), 21.7 (MnO, 1.5 T), 296.11 (MnFe, 1.5 T)	t_1/2_ = 59.76 min (MnO), 63.04 min (Mn_3_O_4_), 2.5 h (MnO_2_), >3 h (MnFe_2_O_4_)	[166, 167, 168, 169, 170, 171, 172, 173–174]
Gadolinium‐containing	r_1_ = 5.3 (Gd_2_O_3,_ 1.5 T), 12.51 (gadolinium‐hyaluronic acid NPs, 3 T), 34.3 (Gd complex bound AuNPs, 10 MHz), 35.76 (Gd‐chelated organic NPs, 3 T)	t_1/2_ = 21.6 min (Gd‐based AGuIX NPs), 40 min (Gd_2_O_3_), 6 h (Gd‐chelated organic NPs)	[175, 176, 177, 178, 179–180]
Iron oxide‐based	r_1_ = 1.16 (SPION‐SiO_2_, 1.5 T), 2.32 (UION@RGD@mPEG, 7 T), 3.6 (USPION, citric acid; 3 T), 4.78 (UION‐PO‐PEG, 3 T), 5.3 (SPION, carboxylic acid; 3 T), 11.4 (SPION, dextran; 1.5 T), 12.1 (SPION‐PEG, 1.5 T), 19 (SPION, chitosan; 1.5 T), 19.9 (SPION, carboxy‐silane, 1.5 T) r_2_ = 13.9 (USPION, citric acid; 3 T), 29.25 (UION‐PO‐PEG, 3 T), 60.2 (SPION, dextran; 1.5 T), 65.3 (SPION‐SiO_2,_ 1.5 T), 67.1 (SPION‐PEG, 1.5 T), 80.4 (SPION, carboxy‐silane, 1.5 T), 181.3 (SPION, carboxylic acid; 3 T), 365.9 (SPION, chitosan; 1.5 T)	t_1/2_ = 1.36 h (SPIO core and carbon shell NPs), 3.77 h (polyampholyte‐coated Fe_3_O_4_ NPs), 10 h (phosphatidylcholine‐coated Fe_3_O_4_ nanomicelles), 14.6 h (PEGylated albumin coated IONPs), 12–48 h (USPION‐citric acid)	[181, 182, 183, 184, 185, 186, 187, 188–189]
Ferrites and mixed‐metal spinel	r_1_ = 12.55 (Zn_0.2_Mn_0.8_Fe_2_O_4_, 3 T), 21.5 (CoFe_2_O_4_‐SiO_2_, 60 MHz), 33.1 (MnFe_2_O_4_@SiO_2_@Gd_2_O(CO_3_)_2_, 4.7 T) r_2_ = 55.58 (Zn_0.2_Mn_0.8_Fe_2_O_4_, 3 T), 138.43 (star‐block copolymer micellar nanocomposites with Mn_0.6_Zn_0.4_Fe_2_O_4_, 1.5 T), 142 (CoFe_2_O_4_‐SiO_2_, 60 MHz), 184.1 (MnFe_2_O_4_@Fe_3_O_4_, 9.4 T), 274 (MnFe_2_O_4_@SiO_2_@Gd_2_O(CO_3_)_2_, 4.7 T)	t_1/2_ > 2 days (Ni_*x*_Fe_2–x_O_3_–PDMAcoAA) t_1/2_ > 2 h (polymer‐encapsulated ZnFe_2_O_4_‐NH_2_)	[190, 191, 192, 193, 194, 195–196]

### 8.1 Manganese‐Based Nanoparticles

Manganese‐based nanoparticles constitute one of the most widely investigated families of T_1_ agents due to the intrinsic paramagnetic character of Mn^2+^, which possesses five unpaired electrons (S = 5/2), thus providing strong dipolar interactions with nearby protons. In nanoscale systems, manganese ions can be incorporated into various crystalline lattices—such as MnO, Mn_3_O_4_, Mn_2_O_3_, MnFe_2_O_4_ or hybrid manganese ferrites—each with distinct relaxation dynamics, electron spin–lattice coupling, and surface accessibility [197]. Among these, MnO nanoparticles, in particular, have drawn attention because the Mn^2+^ oxidation state exhibits fast electronic relaxation, leading to substantial increases in r_1_ relaxivity while maintaining favorable biocompatibility. Furthermore, nanoscale MnO systems enable precise engineering of core dimensions, crystallinity, and aqueous dispersibility, which directly influence water–particle interactions and, consequently, proton relaxation.

Recent advances have focused on hybrid nanoparticles that optimize performance at ultrahigh magnetic fields (≥7 T), which are increasingly used in advanced neuroimaging, preclinical oncology, and functional MRI research. Cheng et al. developed Ho_2_O_3_/MnO_2_ hybrid nanoparticles functionalized with BSA and chlorin e6 (Ce6), resulting in a sophisticated theranostic system capable of delivering combined T_1_–T_2_ contrast and multimodal therapeutic effects [198]. Under acidic tumor microenvironment conditions, MnO_2_ decomposes, releasing Mn^2+^ and activating T_1_ contrast, while the Ho_2_O_3_ core generates strong T_2_ contrast at ultrahigh fields due to its large magnetic moment and pronounced spin–lattice interactions. These nanoparticles also display catalase‐like activity, decomposing H_2_O_2_ to produce O_2_ for alleviating tumor hypoxia, enhancing the efficacy of PDT and radiotherapy (RT). Tumor suppression in vivo is markedly improved relative to single‐mode agents, demonstrating the therapeutic relevance of dual‐mode nanomaterials.

### 8.2 Gadolinium‐Containing Nanostructures (Hybrid Gd‐NPs)

Although not magnetic nanoparticles in the strict sense, gadolinium‐based nanostructures merit brief mention due to their increasing relevance as safer alternatives to small‐molecule Gd chelates. Dydak et al. introduced Gd‐loaded lipid nanoassemblies composed of glyceryl monooleate (GMO) and DTPA‐bis(stearylamide)‐Gd, yielding high relaxivity values (r_1_ ≈ 19.7 mM^−1^s^−1^) [199]. Structural transitions modulated by Gd‐lipid concentration altered water accessibility but maintained high imaging performance. These systems represent a conceptual transition toward nanoparticle‐mediated Gd delivery, even if they remain distinct from the iron and manganese platforms that dominate nanomagnetic MRI.

### 8.3 Superparamagnetic Iron Oxide Nanoparticles (SPIONs)

SPIONs such as Fe_3_O_4_ and γ‐Fe_2_O_3_ demonstrate high r_2_ relaxivities (100–510 mM^−1^s^−1^), making them ideal for T_2_‐weighted imaging in applications such as liver imaging, lymph node staging, and inflammatory lesion detection [200].

Historically, formulations such as Feridex/Endorem, Resovist, and ferumoxtran‐10 (Sinerem/Combidex) have demonstrated high diagnostic sensitivity. However, only ferumoxytol remains widely used today. Ferumoxytol (Feraheme), approved for treating iron deficiency anemia, has become a cornerstone in various MRI protocols due to its long circulation time (>14 h), high molecular stability, and consistent T_2_/T_2_* performance.

Recent clinical work has further validated SPIONs as powerful diagnostic tools for i) liver cancer and metastasis, where SPION‐enhanced MRI provides ≥90%–95% sensitivity for small hepatocellular carcinoma nodules in patients with cirrhosis, with superior tumor margin definition compared to Gd‐based agents (2023–2024 studies); ii) lymph node staging, where Ferumoxtran‐10 provides diagnostic accuracies between 89% and 92% in prostate and pancreatic cancer, dramatically reducing unnecessary lymphadenectomies (Phase III trials NCT04261777, 2024–2025); and iii) neuro‐oncology, where Ferumoxytol improves ability for differentiating between tumor recurrence and pseudoprogression, with markedly better vascular leakage characterization compared to Gd chelates (NCT00103038, NCT01973517).

The integration of magnetic nanoparticles with nuclear imaging isotopes has enabled hybrid systems capable of providing both structural (MRI) and functional (PET/SPECT) data from a single nanoplatform. Xi et al. developed an RGD‐conjugated SPION nanoprobe functionalized with DTPA chelators capable of binding radioisotopes such as ^99m^Tc for SPECT imaging [201]. In 4T1 tumor‐bearing mice models, the nanoprobe showed high tumor accumulation mediated by α_v_β_3_ integrin targeting, strong T_2_‐weighted contrast at the lesion site, and precise radiotracer‐based mapping of tumor metabolism. Such multimodal platforms exemplify the increasing convergence between molecular imaging and anatomical MRI, offering a comprehensive diagnostic toolkit with direct translational potential.

### 8.4 Ultrasmall Iron Oxide Nanoparticles (USPIONs)

Iron oxide nanoparticles have historically been considered T_2_ agents due to their strong superparamagnetic effect. However, when engineered at ultrasmall sizes (≤5 nm), they exhibit markedly different magnetic behavior, resulting in T_1_‐dominant relaxivity profiles [202]. USPIONs benefit from high surface‐area‐to‐volume ratios, allowing more efficient water proton exchange. Because their magnetic moment scales nonlinearly with diameter, reducing the core size diminishes transverse dephasing effects, thereby increasing the r_1_/r_2_ balance and enabling bright, T_1_‐weighted signal enhancement.

One of the most robust demonstrations of USPION clinical potential was provided by Besenhard et al., who developed a continuous‐flow aqueous synthesis method capable of producing monodisperse ∼5 nm iron oxide nanoparticles at large scale (>15 L/day) [203]. These USPIONs exhibited r_1_ values exceeding 10 mM^−1^s^−1^ and r_2_/r_1_ ratios ≤4, matching or surpassing the performance of state‐of‐the‐art gadolinium‐free T_1_ agents. Their high colloidal stability, reproducibility across batches, and compatibility with hydrophilic coatings such as dextran, PEG, and zwitterionic polymers position them as excellent candidates for clinical translation.

Hybrid USPION platforms have also been developed to enhance dual T_1_/T_2_ responsiveness. Beck et al. synthesized USPIONs coated with carboxymethyl‐dextran (CMDex), achieving combined T_1_ and T_2_ contrast with r_1_ = 0.17 mM^−1^s^−1^ and r_2_ = 1.73 mM^−1^s^−1^ at 7 T (r_2_/r_1_ = 10) [204]. These nanoparticles demonstrated clear T_1_ signal enhancement at low concentrations (≥1.7 mM Fe) and strong T_2_ darkening at higher concentrations, confirming their suitability for applications requiring multi‐contrast. Toxicity assessments showed >90% viability across several cell lines, reinforcing their biocompatibility.

### 8.5 Ferrites and Mixed‐Metal Spinel Nanoparticles

Among hybrid magnetic systems, mixed‐metal ferrites—such as zinc–manganese ferrites and manganese–ferrite spinels—have emerged as some of the most effective dual‐mode platforms due to their tunable magnetic anisotropy, high crystallinity, and adjustable electron spin configurations [205]. The incorporation of Mn^2+^ into spinel structures modulates electron relaxation rates while increasing saturation magnetization, enabling simultaneous enhancement of both r_1_ and r_2_ relaxivities. Rezaei et al. demonstrated that Zn_0.__3_Mn_0.__5_Fe_2.__2_O_4_ nanoparticles coated with PEG exhibited the most balanced MRI performance across tested ferrite compositions, producing robust T_1_ and T_2_ signal modulation while maintaining favorable biocompatibility and colloidal stability [206]. Importantly, the r_2_/r_1_ ratio in these systems remains within a range compatible with dual‐mode imaging, avoiding the suppression of T_1_ contrast commonly observed in large‐magnetization T_2_ agents.

### 8.6 Stimuli‐Responsive Nanoparticles

**FIGURE 10:**
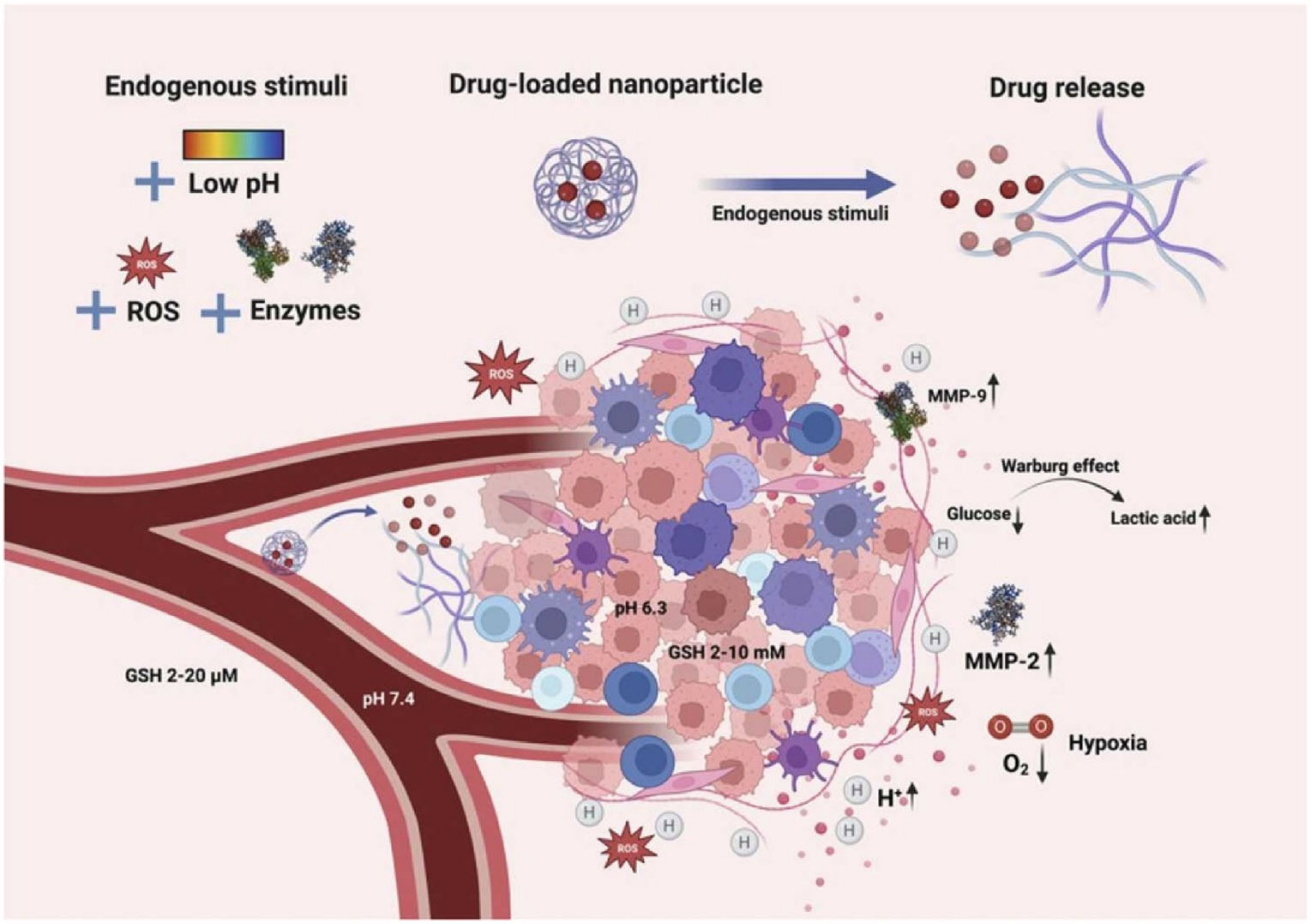
Endogenous stimuli‐responsive nanoparticles for cancer therapy. Reproduced with permission from Xie et al. [208]. Copyright 2022, Elsevier.

The emergence of stimuli‐responsive nanoparticles has substantially expanded the functional possibilities for MRI. These nanosystems exploit pathological cues—such as pH, redox gradients, hypoxia, enzymatic activity, or elevated H_2_O_2_ levels—to trigger dynamic alterations in particle structure, magnetic behavior, ion, or drug release (Figure 10). In 2022, Lu et al. introduced SPIO@SiO_2_@MnO_2_ nanoparticles, an elegant demonstration of pH‐responsive contrast switching [207]. Under physiological conditions, the silica layer and intact MnO_2_ shell suppress both T_1_ and T_2_ signals, minimizing background noise. However, in acidic environments typical of tumors or inflammatory tissues, the MnO_2_ shell decomposes, releasing Mn^2+^ ions that produce strong T_1_ contrast. Simultaneously, dissolution exposes the underlying SPIO core, restoring T_2_ functionality. This sequential activation produces a 12.3‐fold enhancement in double‐contrast subtraction imaging, enabling the characterization of liver metastases and visualization of inflammatory lesions.

Another compelling example is the tumor‐hypoxia–responsive Fe_3_O_4_‐Met‐Cy5.5 nanoprobe developed by Yang et al., which incorporates a metronidazole moiety that undergoes reductive activation in hypoxic conditions [209]. This selective reduction enhances retention in hypoxic tumor regions, producing strong T_1_ contrast at 6 h post‐injection and maintaining signals for up to 24 h. Tissue regions in MRI images colocalize with HIF‐1α–positive zones, illustrating the capacity of redox‐responsive systems for mapping physiologically relevant gradients within tumors.

### 8.7 Surface Engineering and Functionalization

**FIGURE 11:**
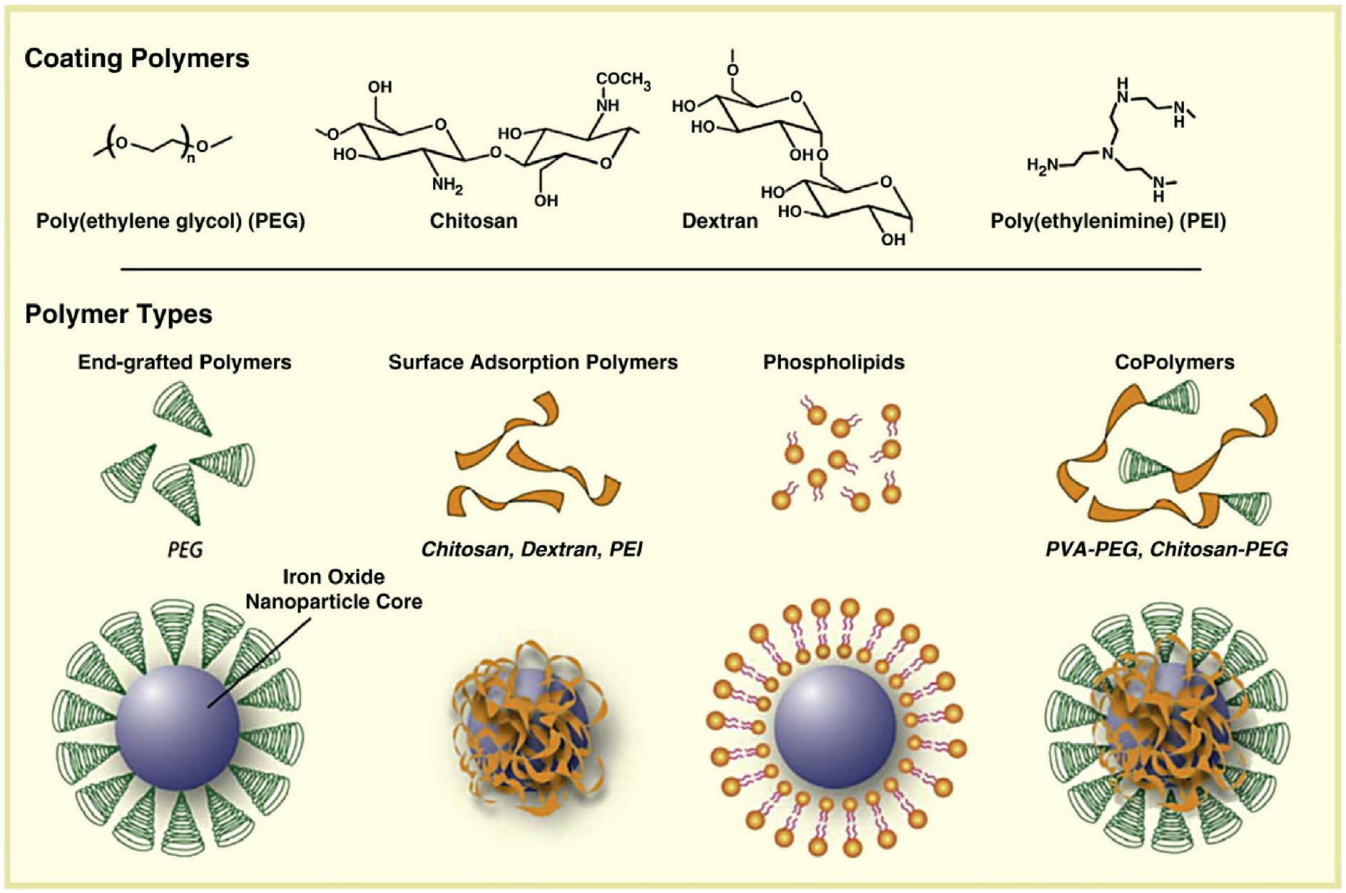
Commonly used molecules for functionalizing magnetic nanoparticles. Reproduced with permission from Veiseh et al. [211]. Copyright 2009, Elsevier.

The surface chemistry of magnetic nanoparticles is one of the most important factors governing their biological performance as MRI CAs, influencing colloidal stability, circulation half‐life, biodistribution, cellular uptake, and ultimately the relaxivity profile observed in vivo. Bare magnetic cores—whether composed of Fe_3_O_4_, γ‐Fe_2_O_3_, MnO, Mn_3_O_4_, or mixed‐metal ferrites—are generally hydrophobic, prone to aggregation, and susceptible to oxidation or ion leaching under physiological conditions. This necessitates the incorporation of surface coatings for stabilization and for ensuring biocompatibility and consistent magnetic behavior (Figure 11) [3]. Such coatings create a protective barrier that modulates water access to the magnetic core, regulating proton exchange, and minimizing recognition by serum proteins, thereby preventing rapid clearance by the MPS [210].

Hydrophilic polymer coatings, including PEG, Dex, CMDex, polyvinyl alcohol (PVA), and zwitterionic polymers, remain among the most widely used, for enhancing stability and prolonging circulation time. PEGylation, in particular, reduces opsonization by plasma proteins and decreases uptake by Kupffer cells, thereby improving tumor accumulation *via* the EPR effect [212]. The molecular weight, grafting density, and conformational flexibility of PEG chains significantly influence the hydrodynamic size and stealth behavior of nanoparticles, with dense or brush‐like PEG architectures providing superior steric repulsion and reduced protein corona formation. Dextran‐based coatings, historically used in formulations such as Feridex and Resovist, not only stabilize the magnetic core but also increase water accessibility, which can enhance r_1_ relaxivity in USPIONs.

Inorganic coatings, especially silica, offer unique advantages in terms of mechanical stability, chemical inertness, and tunable porosity. Silica layers can be deposited as dense shells that regulate water diffusion or engineered as mesoporous structures that allow controlled loading of therapeutic agents, fluorescent dyes, or radionuclides for multimodal imaging. The SPIO@SiO_2_@MnO_2_ system described by Lu et al. demonstrated the sophisticated use of inorganic coatings for achieving pH‐responsive behavior, where silica acts as a diffusion barrier under physiological conditions but becomes permeable upon MnO_2_ decomposition in acidic tumor microenvironments, enabling sequential activation of T_1_ and T_2_ contrast [207]. Silica‐coated ferrites also provide favorable platforms for conjugation with peptides, antibodies, and small‐molecule ligands due to the ease of surface silanization reactions.

Phospholipid and biomembrane‐mimetic coatings have emerged as promising alternatives for applications requiring high biocompatibility and minimal immune activation. Lipid bilayers can encapsulate magnetic cores to form magneto‐liposomes, improving serum stability and enabling the codelivery of chemotherapeutic agents or photosensitizers. Dydak et al. employed lipid nanoassemblies to deliver Gd‐based agents with enhanced relaxivity, but similar architectures are readily applicable to Fe‐ and Mn‐based nanoparticles [199]. Moreover, hybrid lipid–polymer systems allow the incorporation of targeting ligands such as folate, transferrin, RGD peptides, HER2 antibodies, and aptamers, thereby enabling active receptor‐mediated uptake in tumor tissues or angiogenic vasculature [213].

Surface charge plays a critical role in regulating cellular interactions and biodistribution. Highly positive surfaces tend to promote nonspecific protein adsorption and rapid macrophage uptake, whereas strongly negative surfaces may exhibit poor cellular internalization. Neutral or zwitterionic coatings often achieve the longest circulation times due to minimal electrostatic interactions with plasma components. Nevertheless, localized positive charge densities can be intentionally introduced to enhance uptake in specific tissues or subcellular compartments when combined with cleavable linkers that dissociate under pathological conditions such as low pH or reductive stress.

Another crucial aspect of surface engineering involves controlling the thickness and permeability of the coating. For T_1_ agents such as MnO‐based nanoparticles and USPIONs, water accessibility to the magnetic core is essential for maximizing r_1_ relaxivity. In this regard, thin hydrophilic coatings that allow efficient proton exchange are preferred. Conversely, T_2_ agents benefit from coatings that preserve magnetic moment and minimize dipolar relaxation damping. For dual‐mode agents, core–shell architectures must be designed with exquisite precision to balance competing relaxation mechanisms. In Zn–Mn–Fe spinel ferrites and HoMn hybrid systems, optimized coating thickness ensures that both Mn^2+^ ion release and magnetic susceptibility contributions are preserved, enabling sequential or simultaneous T_1_–T_2_ contrast depending on the biological environment [198].

The protein corona formed upon exposure of nanoparticles to serum proteins significantly alters nanoparticle identity, influencing biodistribution, relaxation behavior, and targeting accuracy. The composition of the corona depends on surface chemistry, hydrophobicity, charge, and curvature and can either hinder or enhance performance. Advanced engineering approaches aim to control corona formation using precoating strategies, “corona‐resistant” polymers, or preadsorption of selected proteins to create a “biomimetic” corona that enhances biological compatibility and targeting specificity.

Collectively, surface engineering plays a fundamental role in transforming magnetic nanoparticles from simple magnetic cores into sophisticated biomedical tools capable of delivering predictable, reproducible, and highly sensitive MRI contrast across diverse fields.

### 8.8 Toxicity, Biocompatibility, and Clearance

The translation of magnetic nanoparticles into clinically viable MRI CAs requires a comprehensive understanding of their toxicity, biocompatibility, pharmacokinetics, and clearance pathways. Unlike small‐molecule gadolinium chelates, whose toxicity profile is governed primarily by renal excretion and chelate stability, magnetic nanoparticles interact with multiple biological systems simultaneously, including the MPS, serum proteins, hepatobiliary pathways, and intracellular degradation components. These interactions depend strongly on nanoparticle composition, size, surface chemistry, aggregation state, and biodegradability, making toxicological evaluation a central component of their development.

Manganese‐based nanoparticles present unique toxicity concerns related to the potential release of free Mn^2+^ ions, which are known to induce neurotoxicity, oxidative stress, and disturbances in calcium homeostasis. In vivo studies have demonstrated that prolonged or uncontrolled Mn^2+^ exposure may cause motor deficits, blood–brain barrier compromise, cerebral edema, and neuronal degeneration, as observed in rodents exposed to MnO nanoparticles for extended periods [214]. Johnston et al. provided a critical comparative analysis of commercially available Mn_2_O_3_ nanoparticles from different manufacturers, revealing consistent cytotoxicity driven by ROS generation and caspase‐3 activation, even in the absence of significant pro‐inflammatory responses mediated by caspase‐1 [215]. These findings underscore the need for precise material characterization and stringent surface functionalization strategies when designing manganese‐based T_1_ MRI CAs.

Surface coatings play a decisive role in mitigating Mn‐based toxicity. PEG, dextran, phospholipids, BSA, silica, and polymer–lipid hybrid shells can significantly reduce the release of Mn^2+^, stabilizing the nanoparticle surface under physiological conditions, and limiting nonspecific uptake by macrophages and Kupffer cells in the liver. Properly engineered coatings delay biodegradation and modulate proton accessibility to the magnetic core, thereby balancing safety with imaging performance. Nevertheless, the long‐term fate of Mn‐based nanoparticles remains an active area of investigation, particularly regarding the kinetics of Mn^2+^ liberation in acidic lysosomal environments and subsequent systemic redistribution.

In contrast, iron‐based nanoparticles—particularly Fe_3_O_4_ and γ‐Fe_2_O_3_ formulations—generally exhibit favorable biocompatibility, largely because iron is an endogenous element incorporated into natural metabolic pathways involving ferritin, transferrin, and heme synthesis [216]. Upon cellular uptake, SPIONs are typically trafficked to lysosomes, where the acidic environment facilitates gradual dissolution of the iron oxide core and incorporation of released Fe^2+^/Fe^3+^ into ferritin or transferrin pools. This natural assimilation contributes to the safety of iron‐based MRI CAs and has contributed to the clinical success of formulations such as ferumoxytol. However, excessive or repeated dosing may still result in transient iron overload, oxidative stress, or altered macrophage function, particularly in individuals with dysregulated iron homeostasis. For this reason, careful dose optimization and long‐term monitoring remain essential for translational applications.

Nanoparticle size strongly influences biodistribution and clearance. Hydrodynamic diameters below 5–6 nm may allow partial renal filtration, whereas larger nanoparticles (10–100 nm) are more likely to undergo hepatobiliary clearance or accumulate within the MPS. Particles above ∼200 nm are rapidly sequestered by macrophages and splenic filtration mechanisms, drastically reducing circulation time and limiting imaging utility. USPIONs, with core sizes ≤5 nm and hydrodynamic diameters maintained below ∼20 nm via thin hydrophilic coatings, demonstrate prolonged circulation times and reduced MPS uptake, making them particularly attractive as T_1_ agents [217]. Conversely, larger SPIONs designed for T_2_ imaging rely on controlled MPS uptake for liver and lymph node imaging, illustrating that the desired toxicity profile is application dependent.

The formation of a protein corona adds another layer of biological complexity. Once in contact with blood components, nanoparticles rapidly adsorb proteins such as albumin, apolipoproteins, fibrinogen, and complement factors. This adsorbed layer effectively redefines the biological identity of the nanoparticle, influencing cellular interactions, complementing activation, clearance kinetics, and even relaxivity patterns. Strategies to control or exploit the protein corona—such as using zwitterionic coatings, preconditioning with specific plasma proteins, or designing “corona‐resistant” surfaces—are becoming increasingly important for predictable in vivo behavior and reduced off‐target accumulation [218].

Clearance mechanisms differ substantially among nanoparticle classes. Iron oxide nanoparticles are primarily degraded intracellularly in lysosomes and subsequently incorporated into iron metabolism pathways. Manganese‐based nanoparticles may release Mn^2+^ during degradation, which is then eliminated via hepatobiliary or renal routes, depending on speciation and complexation with endogenous biomolecules. Hybrid ferrites and rare‐earth systems show more complex behavior, as rare‐earth elements such as holmium or gadolinium may have limited metabolic pathways and therefore require careful assessment of long‐term accumulation, especially in bone and liver tissues [198].

Taken together, toxicity, biocompatibility, and clearance represent interdependent pillars in the successful translation of magnetic nanoparticles into clinically useful MRI CAs. Advances in surface engineering, composition tuning, and controlled biodegradation have significantly improved safety profiles, but rigorous toxicological testing and long‐term biodistribution studies remain essential to ensure that emerging nanomaterials meet regulatory and clinical standards.

### 8.9 Emerging Trends (2022–2025)

In recent years, a rapid expansion has occurred in the conceptual and technological landscape of magnetic nanoparticle–based MRI CAs, driven by advances in nanochemistry, machine learning, biomimetic engineering, and image‐processing methodologies. These developments have not only improved contrast efficiency and biocompatibility but have also opened new diagnostic and therapeutic possibilities unimaginable a decade ago. As MRI technology evolves toward higher spatial resolution, quantitative imaging, and compatibility with low‐field systems, magnetic nanoparticles have adapted accordingly, reflecting a shift toward multifunctional, intelligent, and physiologically responsive nanosystems capable of integrating imaging, therapy, and biological sensing.

One of the most transformative trends between 2022 and 2025 is the incorporation of AI into the design, optimization, and interpretation of nanoparticle‐based MRI. Machine learning algorithms have been increasingly employed to predict relaxivity behavior from nanoparticle composition, shape, surface chemistry, and magnetic anisotropy, thereby accelerating discovery and reducing dependence on empirical synthesis [219]. Deep neural networks have also been used to simulate proton–nanoparticle interactions at clinically relevant magnetic fields, predict r_1_ and r_2_ relaxivities under different physiological conditions, and optimize particle geometry for dual‐mode or high‐field applications. On the imaging side, AI‐enhanced reconstruction algorithms allow for the extraction of higher‐quality contrast from lower nanoparticle doses, reducing toxicity risks and enabling safer longitudinal monitoring—an important step toward regulatory acceptance.

Another emerging direction involves the development of biodegradable magnetic nanoparticles, intended to address longstanding regulatory concerns associated with long‐term retention. Traditional SPIONs and manganese oxide nanoparticles rely on slow lysosomal degradation. However, new formulations integrate degradable cores or shells that disassemble into nontoxic components over predictable timescales. Examples include iron‐doped bioresorbable silicates, Mn‐based nanoclusters stabilized by acid‐labile ligands, and organic–inorganic hybrid nanoparticles with enzymatically cleavable coatings. These biodegradable systems aim to combine high relaxivity with controlled clearance, minimizing accumulation in the liver, spleen, and lymphatic tissue, while maintaining sufficient stability during imaging windows.

The renewed interest in low‐field MRI (LF‐MRI) has also influenced nanoparticle design strategies. Whereas high‐field MRI (≥7 T) favors strong T_2_ effects due to enhanced susceptibility‐driven dephasing, low‐field MRI (≤0.5 T) benefits from CAs with high r_1_ values, making T_1_‐active nanoparticles particularly attractive [220, 221]. USPIONs—whose r_2_/r_1_ ratios approach unity at low field strengths—have become strong candidates for portable and point‐of‐care MRI applications. These compact systems, which are less expensive and more accessible in resource‐limited settings, can leverage nanoparticle‐enhanced T_1_ contrast to compensate for reduced inherent tissue contrast in low‐field regimes. Moreover, the reduced susceptibility artifacts at low field enable more accurate quantification of nanoparticle distribution, supporting applications in inflammation monitoring, pediatric imaging, and longitudinal disease tracking. For hyperthermia treatment, the use of higher magnetic field strengths is advantageous as the specific absorption rate increases, providing high heat dissipation (e.g., for cancer cell ablation) and stronger targeting of magnetic nanoparticles. However, at extremely high magnetic fields superparamagnetic nanoparticles tend to agglomerate, which can affect their clinical potential. Ultimately, the choice of magnetic field is application dependent.

Simultaneously, theranostic magnetic systems—which integrate MRI contrast with therapeutic payloads such as chemotherapeutics, immunomodulators, radionuclides, gene editing constructs, or photodynamic agents—have advanced considerably. Hybrid HoMn‐Ce6 nanoparticles described by Cheng et al. exemplify the growing sophistication of these systems [198]. These particles combine MRI responsiveness with catalase‐like activity that alleviates tumor hypoxia and enhances both RT and PDT, representing an increasingly favored strategy that links diagnostics with biological interventions. Similar approaches employ magnetic hyperthermia, ROS‐generating ferrite nanoparticles, and redox‐activated drug release triggered by intracellular glutathione gradients or ROS.

A particularly promising area is the integration of magnetic nanoparticles with biological targeting mechanisms, including biomimetic coatings derived from cell membranes (e.g., erythrocyte, platelet, or cancer‐cell membrane cloaking), exosome‐inspired vesicles, and surface proteins that mimic endogenous recognition cues. These biomimetic approaches are used for significantly prolonging circulation time, evading immune clearance, and for promoting homotypic tumor targeting, thereby improving both accumulation and diagnostic precision. The ability to combine biomimicry with modular surface engineering allows the creation of finely tuned platforms suitable for both imaging and therapeutic applications.

Multimodal magnetic nanoparticles, capable of combining MRI with fluorescence, PAI, PET, SPECT, CT, or ultrasound contrast, continue to gain prominence. These multifunctional systems provide comprehensive anatomical and physiological information, reducing diagnostic uncertainty, and enabling precise guidance for image‐driven interventions. Nanoparticles such as RGD‐functionalized SPIONs radiolabeled for SPECT [222] and SPIO@SiO_2_@MnO_2_ hybrids capable of pH‐triggered T_1_–T_2_ switching are representative examples of how nanoplatforms can unify complementary imaging techniques and contextualize MRI data within broader biological frameworks.

Collectively, the trends emerging between 2022 and 2025 demonstrate a decisive movement toward magnetic nanoparticle platforms that are safer, more intelligent, and more versatile. These developments not only enhance MRI diagnostic power but also pave the way for fully integrated theranostic solutions and widespread adoption in both high‐field and low‐field imaging environments.

Magnetic nanoparticles have evolved into one of the most versatile and promising platforms for contrast enhancement in MRI, offering a level of structural tunability, magnetic responsiveness, and biochemical adaptability far beyond what is achievable with conventional small‐molecule agents. Over the last decade—and in particular between 2022 and 2025—advances in nanocrystal synthesis, surface engineering, and environmental responsiveness have transformed these materials into highly sophisticated diagnostic tools capable of delivering enhanced relaxivity, improved biocompatibility, and precise targeting of pathological microenvironments. The integration of USPIONs, manganese‐based nanostructures, mixed‐metal ferrites, and high‐field hybrid systems has enabled unprecedented control over T_1_, T_2_, and dual‐mode T_1_–T_2_ contrast mechanisms, allowing clinicians and researchers to obtain more reliable and nuanced information from MRI scans.

These developments have been paralleled by major improvements in nanoparticle stability, circulation time, and surface functionality, driven by innovations in polymer coatings, silica shells, phospholipid bilayers, and biomimetic cloaking strategies. Together, these efforts have significantly reduced toxicity risks—particularly those associated with Mn^2+^ release and long‐term iron accumulation—while enabling predictable biodistribution and controlled degradation pathways. The emergence of stimuli‐responsive nanosystems, capable of selectively activating T_1_ or T_2_ contrast in response to physiologically relevant cues such as pH, redox potential, enzymatic activity, or hypoxia, has expanded the diagnostic scope of MRI by providing context‐specific results that correlate with tumor metabolism, inflammation dynamics, and disease progression.

Recent clinical investigations, including studies involving ferumoxytol and ferumoxtran‐10 in oncology and neuroimaging, demonstrate that magnetic nanoparticles are not merely experimental materials but are increasingly integrated into sophisticated diagnostic workflows. Their compatibility with both high‐field and low‐field MRI, combined with the advantages offered by theranostic hybrid systems, suggests that magnetic nanoparticle CAs will occupy an essential role in future precision medicine strategies. The incorporation of AI into nanoparticle design and MRI interpretation further enhances this trajectory, enabling prediction‐driven synthesis, optimized relaxivity frameworks, and accurate diagnostic assessments at lower doses.

Despite these advances, several challenges remain before widespread translation can occur, including regulatory standardization, scalable manufacturing, long‐term toxicological assessment, and the need for harmonized imaging protocols across clinical platforms. Nonetheless, the convergence of nanotechnology, molecular imaging, AI, and theranostics positions magnetic nanoparticles at the forefront of next‐generation MRI innovation. Continued interdisciplinary collaboration will be essential to fully harness the diagnostic and therapeutic potential of these materials, paving the way for safer, more specific, and more powerful imaging tools that can address the complex demands of modern biomedical practice.

## 9 Lipid Nanoparticles as Contrast Agents

Lipid nanoparticles (LNPs) have attracted attention due to their high versatility in biomedical imaging, because of their intrinsic biocompatibility, structural tunability, and ability to encapsulate a broad range of cargos, from small organic dyes to metal‐based CAs and inorganic nanomaterials. Integrating imaging probes into a lipid matrix improves its circulation time and biodistribution, thereby improving both signal quality and safety. These properties make LNPs attractive candidates for enhanced fluorescence imaging (FI) and MRI, and for the integration of multimodal and theranostic methodologies, especially within the domains of oncology and neuroimaging [223, 224].

LNPs comprise of a diverse family of nanostructures, including liposomes, micelles, nanodiscs, cubo/hexosomes, lipoprotein‐based particles, nanoemulsions, and nanobubbles. These NPs are used with various imaging modalities such as MRI, CT, optical, ultrasound, and PAI. They enable multimodal contrast and molecular targeting but still face challenges related to payload access and clinical translation. It is well known that their wide range of morphologies is responsible for modulating loading, circulation, and targeting. Their architecture or morphology controls which imaging payloads can be incorporated and how particles behave in vivo [225, 226, 227–228]. Among them, liposomes are vesicular structures composed of a lipid bilayer and hydrophilic core, able to accommodate amphiphilic agents within the bilayer, and widely used as scaffolds for gadolinium ion (Gd^3+^) chelates, iodinated molecules, and multimodal imaging formulations that combine different contrast mechanisms [229]. Conversely, micelles are smaller amphiphilic aggregates with a hydrophobic core and hydrophilic shell, making them ideal for solubilizing lipophilic CAs and enabling faster tissue penetration compared with liposomes. Nanocrystal micelles and lipid‐coated particles are important variations, in which a lipid shell surrounds inorganic cores, such as iron oxide, gold, or quantum dots, allowing aqueous solubilization and surface functionalization for multimodal imaging applications.

Furthermore, nanodiscs are modular, discoidal lipid assemblies that solubilize lipophilic magnetic resonance (MR) agents and enable straightforward conjugation of additional labels, thus supporting cellular imaging that combines MR and optical modalities [230]. Cubic and hexagonal lipid phases (cubosomes and hexosomes) provide highly organized internal nanoenvironments for embedding paramagnetic nitroxide lipids, yielding high‐relaxivity CAs for MR imaging [231]. Lipoprotein‐inspired particles, based on HDL‐ or LDL‐based platforms, repurpose endogenous nanoparticles to carry MR, CT, or optical labels, taking advantage of favorable biological interactions and efficient access to atherosclerotic plaques in disease models [232]. In addition, lipid nanoemulsions and iodinated lipid nanodroplets have been developed as CT CAs with high iodine payload, good physicochemical stability, prolonged blood‐pool residence time, and potential for large‐scale manufacturing [233]. Finally, nanobubbles and nanoscale ultrasound CAs, consisting of gas‐core or phase‐change constructs stabilized by lipid shells, have been employed in intra‐ and extra‐vascular ultrasound molecular imaging, as well as in the noninvasive exploration of tumor microenvironments [234].

The wide range of LNPs applications is only possible because of their high biocompatibility, as LNPs are constructed from lipids that resemble or are derived from physiological phospholipids and triglycerides. This structural similarity favors biodegradation through normal lipid metabolic pathways and reduces the risk of immunogenic or idiosyncratic reactions. For instance, Tenchov et al. published a systematic review highlighting the outstanding and versatile applications of LNPs as platforms for drug delivery [235]. Different LNP‐based delivery systems are already under advance investigation for theranostic applications, nucleic acid therapy, and vaccine administration.

The capacity of LNPs for encapsulation and specificity for targeting opens a large number of possibilities for their use as imaging agents or diagnostic probes. Due to the capacity of LNPs to encapsulate and deliver therapeutics and imaging agents to specific sites, LNPs can act as carriers for targeted imaging. LNPs can act as transporters of contrast molecules, making them valuable alternatives in diagnostic imaging. This capability is crucial for differentiating internal body structures or pathological processes during imaging procedures [236]. Compared with some traditional CAs, for example free Gd^3+^‐chelates, LNP‐based systems can lower the effective dose of metal and reduce systemic exposure and retention in healthy tissues [235, 236].

Different CAs, such as dyes and/or inorganic nanoparticles can be encapsulated by LNPs, protecting them from rapid enzymatic degradation, oxidation, and opsonization. Furthermore, encapsulation can limit nonspecific binding to off‐target tissues, by modulating pharmacokinetics, and maintaining an optimal local concentration of the probe at the imaging site. For MRI, the lipid microenvironment can be engineered to fine‐tune the balance between water accessibility (essential for relaxivity) and structural protection of the paramagnetic center [236]. For instance, Kostevšek et al. synthesized a large range of magnetic liposomes (MLs) with iron oxide nanoparticles (i.e., IO‐NPs@MLs embedded in a lipid bilayer), with different phospholipid formulations evaluated to test the relaxivity of the MLs [237]. The potential of MLs as CAs for T24 cancer cells was evaluated through in vitro magnetic resonance measurements. The results suggest a strong correlation between the fluidity of the magnetoliposome (ML) bilayer and their relaxivity (r_2_) values, where the incorporation of 5 nm IO‐NPs significantly increases the r_2_ values from 153 ± 5 mM^−1^s^−1^ for DPPC/cholesterol/DSPE‐PEG to 673 ± 12 mM^−1^s^−1^ for DOPC/DSPE‐PEG, in contrast with IO‐NPs alone (i.e., r_2_ = 16 mM^−1^s^−1^). The MRI results indicate that IO‐NPs@MLs were selectively taken up by T24 cells, increasing contrast and improving the distinction between healthy and neoplastic cell lines. Therefore, careful selection of the lipid bilayer components for MLs can lead to efficient MRI CAs, even at very low IO‐NP concentrations.

Seo et al. showed that modifying LNP surfaces through PEGylation, chitosan coatings, and surfactant‐based shell engineering significantly increases their bioavailability, overcoming limitations associated with delivery, targeting, and selectivity [238]. Furthermore, specific surface modifications, for example with genetically engineered oleosin, improved structural stability, highlighting these features as important in minimizing aggregation and fusion. Due to its multimodal nature, both hydrophobic and hydrophilic agents can be incorporated, such as lipophilic dyes in the bilayer and hydrophilic chelates in the aqueous core. Therefore, LNPs can be designed to support MRI, optical, ultrasound, photoacoustic, or nuclear imaging in a single platform [236, 239].

Selective targeting of ligand‐modified LNPs is one of the major advantages compared with traditional CAs, as the ability to deliver cargo to specific tissues can modulate diagnostic precision by enabling higher local concentrations of CAs in target tissues, such as tumors or inflammatory lesions, reducing background signals. In addition, controlled release from the lipid matrix can be made responsive to pH, enzymes, or temperature, enabling site‐specific release of the encapsulated agent. This feature is crucial at tumor microenvironments, which often show acidic pH, elevated protease activity, and abnormal vasculature [235, 240].

The coencapsulation of different imaging probes, for example Gd^3+^‐chelates and NIR dyes, facilitates the simultaneous assessment of high‐resolution anatomical data derived from MRI and highly sensitive molecular features acquired from optical imaging [239, 241]. By incorporating therapeutic agents into these systems, their capability for theranostics enhances, thereby integrating diagnostic processes, targeted therapeutic interventions, and longitudinal monitoring strategies [240, 242].

### 9.1 Lipid Nanoparticles for FI

Encapsulation of fluorescent dyes in LNPs alters their photophysical behavior due to changes in local polarity, viscosity, and aggregation state. This can suppress self‐quenching, reduce photobleaching, and enhance quantum yield. For instance, Shu et al. showed that DiR‐loaded solid lipid nanoparticles (DiR@SLNs) decrease dye–dye interactions because the dye is predominantly confined to the hydrophobic lipid core in a core–shell architecture [243]. The DiR moieties are confined in the cetyl palmitate core, which is advantageous for bioimaging applications. The lipid environment also shields the chromophore from aqueous quenchers and ROS. As a result, DiR‐SLNs showed high physicochemical stability, maintaining ∼90% of their fluorescence intensity over 10 days, indicating not only high photostability but also structural integrity of the nanoparticle during storage and in biological fluids. The near‐infrared window also minimizes tissue autofluorescence and light scattering, enabling deep‐tissue bioimaging.

Indocyanine green (ICG) is a clinically approved near‐infrared fluorescent (NIRF) dye for tumor targeting but suffers from rapid degradation, aggregation, and nonspecific distribution when used alone. Incorporating ICG into LNPs (ICG‐LNPs) improves its photostability and quantum yield by isolating the dye within the lipid phase and limiting unfavorable aggregation. Wu et al. demonstrated that ICG‐loaded liposomes could be used for photoacoustic CT, for evaluating in vivo tumor penetration of liposomal nanocarriers [244]. Prasad et al. developed ICG‐encapsulated, targetable LNPs (ICG@LNPs) to improve performance as CAs for solid tumor imaging [245]. By employing a nanoprecipitation method, they obtained ICG@LNPs with improved characteristics, including high brightness and an enhanced quantum yield of 3.5% in aqueous media, demonstrating optically stable properties over 30 days. This indicates their potential for long‐term imaging applications and chronic disease monitoring. Furthermore, the rapid accumulation (∼1 h) and prolonged retention of the nanoparticles at the tumor site (>168 h) suggest that they could reduce the need for multiple doses, thereby improving patient comfort. Collectively, these examples show how the lipid matrix can be used to tailor the microenvironment of fluorescent probes, improving brightness, stability, and pharmacokinetics without altering the probe's fundamental photophysical properties.

### 9.2 Lipid Nanoparticles for MRI

LNPs are capable of encapsulating a considerable amount of imaging agents, such as CAs used in MRI, which possess intrinsic magnetic properties. For instance, Kostevšek et al. evaluated different SPION‐containing magneto‐liposomes (SPIONs‐MLs), showing their influence on relaxivity (r_2_) values compared with “free” SPIONs [237]. Embedding 5 nm SPIONs in the lipid bilayer resulted in a substantial increase in relaxivity, with r_2_ values reaching up to 673 ± 12 mM^−1^s^−1^ for specific formulations, compared to only ∼16 mM^−1^s^−1^ for the free SPIONs. Additionally, in vitro MRI measurements demonstrated that MLs were selectively taken up by cancerous T24 cells, improving contrast and facilitating the distinction between healthy and cancerous cells. Thus, when SPIONs are embedded in lipid bilayers or nanoemulsions they are able to enhance important physicochemical features as their colloidal stability, dispersity, and biointerface properties are improved, which are crucial for imaging applications.

Manganese oxide–lipid liquid crystalline nanoparticles (LLCNPs@MnO) are another promising system, as manganese ions (Mn^2+^), due to their paramagnetic properties, are suitable for T_1_‐weighted MRI. Flak et al. developed this novel contrast agent, in which manganese oxide nanodomains are embedded in a lipid liquid‐crystalline scaffold [246]. The lipid matrix stabilizes MnO, controls particle size, and provides a biocompatible interface with biological fluids. This architecture enhances the effective correlation time for Mn–water interactions, through restricted rotation and organized water layers at the interface, leading to improved T_1_ relaxivity and robust MRI contrast. The findings suggest that the combination of manganese oxide and lipids could drive innovative advancements in MRI technology.

Manganese can also be associated with ferrite to yield MnFe_2_O_4_ (MFO) nanoparticles functionalized with vitamin E, which were encapsulated into biodegradable and nontoxic nanoemulsions, resulting in VitE‐MFO‐NEs. The most important feature, observed by Díez‐Villares et al., was that the encapsulation process maintained the superparamagnetic properties of the MFO NPs [247]. This novel MRI agent exhibited an extremely high transverse relaxivity of 652.9 × 10^−3^ M^−1^s^−1^, which is approximately twofold higher than the relaxivity of unencapsulated MnFe_2_O_4_. Lipidic encapsulation significantly improved MRI sensitivity by enhancing negative MR contrast, showcasing the potential of these systems as effective CAs. Furthermore, the nanoemulsions demonstrated excellent in vivo biocompatibility, indicating their safety for biomedical applications, particularly as strong contrast enhancers for soft tissues and tumors.

It is well documented in the literature that gadolinium (Gd^3+^) is widely used as a T_1_ contrast agent when chelated. In systems such as GMO@DTPA‐BSA‐Gd, Gd‐chelates are anchored within or onto a lipid nanoparticle, leading to safer Gd‐liposome formulations. For example, Šimečková et al. evaluated the safety of gadolinium‐labeled liposomes (Gd‐lip) in human liver cells and macrophages, finding no cytotoxicity in various liver cell types, including cancerous and differentiated cells [248]. No potential side effects or pro‐inflammatory responses (i.e., NLRP3 activation in HepaRG cells) were detected, suggesting in vivo safety. Dydak et al. showed that GMO@DTPA‐BSA‐Gd are novel and promising MRI CAs [199]. Several physicochemical advantages were highlighted such as (i) the high molecular weight of these systems which can reduce molecular reorientation, enhancing the stability and effectiveness of the CAs during imaging procedures; (ii) the lipid–protein shell which provides multiple water‐accessible sites while keeping Gd tightly chelated, thus improving safety profiles due to their unique composition and potentially reducing adverse effects associated with traditional CAs; and (iii) limited direct exposure of tissues to free Gd^3+^, potentially reducing long‐term retention and toxicity. The use of Gd^3+^‐chelating lipids in MRI could expand the clinical applications of MRI, allowing better diagnosis and monitoring of various medical conditions and opening novel directions toward further innovations in contrast agent technology. LNPs represent integrated, advanced, and versatile platforms for enhancing biomedical imaging through precise control over microenvironments and targeting. Their frequent use is linked to their ability to address the main limitations of conventional CAs. However, their clinical application still requires overcoming challenges related to stability, large‐scale manufacturing, biodistribution, and rigorous validation. Future research should focus on optimized formulation design and on the integration of LNPs with advanced and multimodal imaging techniques.

### 9.3 Lipid Nanoparticles for Nuclear Imaging

Radiolabeled LNPs can be used for deep tissue imaging with high sensitivity and specificity, using PET and SPECT for image‐guided therapy. Varela‐Fernández et al. developed and investigated lactoferrin‐loaded nanostructured lipid carriers (NLCs) as a new formulation for ocular drug delivery [249]. Ocular biopermanence of NLCs was carried out using 2‐[^18^F]‐fluoro‐2‐deoxy‐D‐glucose (^18^F‐FDG) as a radiotracer, with radioactivity evaluation using PET. A higher mean residence time (i.e., > 2×) and half‐life (i.e., >6×) was detected for the NLCs, compared to the standard solution (i.e., containing ^18^F‐FDG). The results reveal that the NLCs exhibit appropriate mucoadhesive, direct cell uptake, and corneal penetration. Mucoadhesive properties were confirmed, with the presence of electrostatic forces for, at least, 240 min, with no evidence of tissue cytotoxicity. In addition, high encapsulation efficiency and loading capacity were obtained (i.e., up to 75%) for effective treatment.

Liposomes can be radiolabeled using ^99m^Tc‐d, lHMPAO, with radiochemical purity greater than 95%. Biodistribution studies and autoradiography studies by Nabar et al. showed significantly higher accumulation of ^99m^Tc labeled stealth liposomes in liver, pancreas, and ascitic fluid of tumor‐bearing mice, compared to normal mice [250]. The ratio of accumulation of radiolabeled liposomes in pancreatic tumors and in normal pancreas was calculated to be very high (i.e., ∼3.5 times higher). Hence, these liposomes offer the possibility to be used as carriers for chemotherapeutic drugs for treatment of pancreatic tumors. The ^99m^Tc‐labeled liposomes showed significant uptake in pancreatic tumors at 3 h. Results indicate that the prepared ^99m^Tc‐liposomes can be useful for diagnostic imaging using SPECT. Tagging these stealth liposomes with different radionuclides such as ^67^Ga, ^177^Lu, and ^111^In may be useful for therapy.

## 10 Conclusions

Many organic, inorganic, and hybrid materials can be used as CAs for optical, magnetic, nuclear, and X‐ray based imaging due to their high absorption, fluorescence, scattering, and relaxivity. Notably, CAs made of heavy metals exhibit high X‐ray attenuation and light absorption, while superparamagnetic and ultrasmall metal oxide nanoparticles exhibit enhanced T_1_ and T_2_ contrast as a result of precise control over size, morphology, and surface chemistry. Other materials that do not possess the intrinsic properties for contrast in bioimaging such as mesoporous materials and lipid nanoparticles can be modified or functionalized to carry cargo for multimodal and targeted imaging. Hybrid nanoparticle architectures, including core–shell systems and nanocomposites, combining magnetic cores with mesoporous silica, zeolites, or MOFs, have emerged as effective systems in overcoming limitations of conventional CAs.

Traditional CAs, usually with low molecular weights, have several limitations, including a short blood circulation time, nonspecific distribution within the body, rapid elimination, significant toxicity, and reduced contrast effectiveness in patients. The incorporation of nanostructured materials into medical imaging and treatment represents a significant shift in the way diseases are diagnosed and treated, moving towards more proactive, personalized, and highly precise interventions. Advances in synthesis and functionalization of nanoparticles have led to improvements in stability, circulation time, and biocompatibility of molecular‐scale and nanosized CAs, often superior to commercial counterparts. Their multifunctional properties, combined with breakthroughs in imaging technologies, have the potential to improve both diagnostic precision and treatment effectiveness. For example, when nanoparticles have accumulated at the disease site, they can be activated by external energy sources (e.g., laser irradiation, alternating magnetic field) for localized treatment, through generation of heat (i.e., hyperthermia) and/or ROS (e.g., from loaded photosensitizers). Combining diagnostic imaging and therapeutic agent delivery into a single system allows for simultaneous detection, treatment, and real‐time monitoring of diseases. Hybrid platforms with enhanced magnetic relaxivity enable controlled water exchange and ion confinement and support multimodal imaging while offering tunable structural and surface properties for targeted and stimuli‐responsive applications. Stimuli‐responsive nanoparticles release significant amounts of therapeutic/imaging cargo only when triggered by specific internal (e.g., pH, enzymes) and/or external (e.g., light, heat, magnetic fields) stimuli at pathological sites, providing “on‐demand” delivery and minimizing systemic toxicity. Ongoing research and development in these areas are essential to overcome current challenges and fully realize the potential of nanostructured CAs in clinical applications.

Despite promising results from in vitro and in vivo experiments, important challenges still limit the clinical translation of nanosized CAs. Among these, the need for a comprehensive assessment of long‐term toxicity, in vivo biodegradation pathways, metal ion release, and interactions with the immune system stands out, as well as the current lack of standardized protocols for physicochemical characterization and preclinical evaluation. The potential toxicity of nanostructures is a concern, as their safety profiles can significantly differ based on their physical properties. The regulatory approval process for nanostructured materials often takes long due to the rigorous procedures applied to ensure the safety, effectiveness and reproducibility of the resulting product. There is still a lot of skepticism and concerns associated with their use by a majority of the public that is likely due to the low number of clinically approved formulations containing nanoparticles. There is a need for awareness with the public and healthcare professionals of the benefits and safety of nanostructured materials, for their acceptance and adoption in the medical field. Overcoming these limitations will require coordinated interdisciplinary efforts, integrating materials science, chemistry, biology, physics, and regulatory science. In short, the findings discussed in this review highlight the great potential of many small molecules and nanomaterials as next‐generation CAs, capable of enabling safer, more sensitive, and personalized diagnostic imaging in modern clinical practice.

## Author Contributions

**Donald Fernandes**: formal analysis. All authors contributed equally to this work. All authors participated in the selection and evaluation of the literature, writing, and full revision of both the initial and final versions of the article.

## Funding

This study was supported by Coordenação de Aperfeiçoamento de Pessoal de Nível Superior (88881.691838, 88881.707398).

## Conflicts of Interest

The authors declare no conflicts of interest.

## Data Availability

All data supporting this review are derived from previously published studies and properly cited throughout the article.
